# Rational design of ^13^C-labeling experiments for metabolic flux analysis in mammalian cells

**DOI:** 10.1186/1752-0509-6-43

**Published:** 2012-05-16

**Authors:** Scott B Crown, Woo Suk Ahn, Maciek R Antoniewicz

**Affiliations:** 1 Department of Chemical and Biomolecular Engineering, Metabolic Engineering and Systems Biology Laboratory, University of Delaware, Newark, DE, 19716, USA

**Keywords:** Metabolic flux analysis, Stable-isotope tracers, Experiment design, Pathway analysis, Statistical analysis, Confidence intervals, Mammalian cells, Free fluxes, Mass spectrometry

## Abstract

**Background:**

^13^C-Metabolic flux analysis (^13^C-MFA) is a standard technique to probe cellular metabolism and elucidate *in vivo* metabolic fluxes. ^13^C-Tracer selection is an important step in conducting ^13^C-MFA, however, current methods are restricted to trial-and-error approaches, which commonly focus on an arbitrary subset of the tracer design space. To systematically probe the complete tracer design space, especially for complex systems such as mammalian cells, there is a pressing need for new rational approaches to identify optimal tracers.

**Results:**

Recently, we introduced a new framework for optimal ^13^C-tracer design based on elementary metabolite units (EMU) decomposition, in which a measured metabolite is decomposed into a linear combination of so-called EMU basis vectors. In this contribution, we applied the EMU method to a realistic network model of mammalian metabolism with lactate as the measured metabolite. The method was used to select optimal tracers for two free fluxes in the system, the oxidative pentose phosphate pathway (oxPPP) flux and anaplerosis by pyruvate carboxylase (PC). Our approach was based on sensitivity analysis of EMU basis vector coefficients with respect to free fluxes. Through efficient grouping of coefficient sensitivities, simple tracer selection rules were derived for high-resolution quantification of the fluxes in the mammalian network model. The approach resulted in a significant reduction of the number of possible tracers and the feasible tracers were evaluated using numerical simulations. Two optimal, novel tracers were identified that have not been previously considered for ^13^C-MFA of mammalian cells, specifically [2,3,4,5,6-^13^C]glucose for elucidating oxPPP flux and [3,4-^13^C]glucose for elucidating PC flux. We demonstrate that ^13^C-glutamine tracers perform poorly in this system in comparison to the optimal glucose tracers.

**Conclusions:**

In this work, we have demonstrated that optimal tracer design does not need to be a pure simulation-based trial-and-error process; rather, rational insights into tracer design can be gained through the application of the EMU basis vector methodology. Using this approach, rational labeling rules can be established *a priori* to guide the selection of optimal ^13^C-tracers for high-resolution flux elucidation in complex metabolic network models.

## Background

^13^C-Metabolic flux analysis (^13^C-MFA) has become a standard tool to probe cellular metabolism and elucidate *in vivo* metabolic fluxes [1-5]. The experimental portion of ^13^C-MFA relies on the introduction of an isotopic tracer (e.g. ^13^C-glucose) to a cell culture, cellular turnover of ^13^C-labeled metabolites through metabolic pathways, and measurement of ^13^C-labeling patterns of metabolites by NMR [6], mass spectrometry (MS) [7-9], or tandem MS [10,11]. The computational portion of ^13^C-MFA relates the measured ^13^C-labeling patterns to metabolic fluxes by iterative least-squares regression. Inherent to successful ^13^C-MFA are high quality measurement data and efficient computer algorithms for simulating isotopomers [12-14], both of which have been investigated in detail. However, an important aspect of ^13^C-MFA that is often overlooked is the selection of appropriate ^13^C-tracers to study a given biological system. ^13^C-Tracers are often selected by convention, or using trial-and-error approaches. With the increasing use of ^13^C-MFA in mammalian systems for therapeutic and industrial applications [15-20], it is surprising to find that literature on the topic of rational tracer selection for ^13^C-MFA is rather limited.

There are many possible choices for ^13^C-tracers in mammalian cultures. Mammalian cells are generally cultured in complex media containing multiple substrates, including glucose, glutamine (and other amino acids), fatty acids and organic acids. Each of these substrates could potentially be selected as the ^13^C-tracer. In the past, ^13^C-glucose, ^13^C-glutamine [21,22], ^13^C-propionate [23,24], and ^13^C-glycerol [25], among others [24-26], have been applied to investigate fluxes in mammalian cells. Currently, the most popular choices are ^13^C-glucose and ^13^C-glutamine tracers, as these compounds are readily metabolized by most mammalian cells. Previously, Metallo et al. [17] evaluated several commercially available ^13^C-glucose and ^13^C-glutamine tracers for studying mammalian metabolism. From a select list of available isotopic tracers, Metallo identified optimal tracers for specific metabolic pathways. Recently, Walther et al. [27] developed a genetic algorithm to optimize mixtures of ^13^C-glucose and ^13^C-glutamine tracers for MFA in mammalian cells. However, because both of these works were based on simulations using a limited number of tracers, they offered no true insights into rational criteria for optimal tracer selection and potentially missed novel and more informative tracers for determining fluxes in mammalian cells.

Central to the ^13^C-tracer experiment design problem are two interconnected issues: 1) how should the optimal tracer be determined; and 2) how should the isotopic experiment be conducted. First, there are many possible tracer substrates commercially available with various ^13^C-labeling patterns. In addition, the possibility to purchase custom synthesized tracers has become a viable option. Moreover, multiple ^13^C-tracers can be applied in a single tracer experiment [28-30], or alternatively, multiple parallel labeling experiments can be performed using a single or multiple ^13^C-tracers [22,31,32]. All of these options increase the complexity of the tracer experiment design space. The number of tracer options quickly increases to a point where it is no longer feasible to efficiently evaluate all possible tracer combinations using simulations and trial-and-error approaches. Therefore, there is a pressing need for new rational approaches for designing tracer experiments to systematically identify optimal tracers, or at least reduce the search space to a computationally more manageable level.

Recently, we introduced a new framework for optimal ^13^C-tracer experiment design based on elementary metabolite units (EMU) decomposition, in which a measured metabolite is decomposed into a linear combination of so-called EMU basis vectors [33]. Our methodology decouples isotopic labeling from flux dependencies in a network model, thus allowing us to draw rational conclusions regarding tracer feasibility, and as such reduce the number of tracer candidates. In this work, we applied the EMU method to a realistic network model of mammalian metabolism, specifically, to the network model proposed by Henry et al. [31] for HEK-293 cell lines with lactate as the measured metabolite. This system is of general interest because it covers all major metabolic pathways of central carbon metabolism and uses an easily accessible extracellular metabolite, i.e. lactate, that is produced by many mammalian cells. The network model of Henry has two free fluxes of interest that must be estimated from ^13^C-labeling data, the oxidative pentose phosphate pathway (oxPPP) flux and pyruvate carboxylase (PC) flux.

In this work, we used the EMU tracer experiment design approach to select optimal tracers in the described system. Our approach is based on sensitivity analysis of EMU basis vector coefficients with respect to free fluxes in the model. Through efficient grouping of coefficient sensitivities, simple tracer selection rules were derived for high resolution of the fluxes in the model. The approach resulted in a significant reduction of the number of possible tracers. The feasible tracers were evaluated using numerical simulations to identify optimal tracers for elucidation of both oxPPP and PC fluxes. The optimal tracers that were identified in this work are novel tracers that have not been previously considered for ^13^C-MFA of mammalian cells; specifically, [2,3,4,5,6-^13^C]glucose for elucidating oxPPP flux and [3,4-^13^C]glucose for elucidating PC flux. We demonstrate that ^13^C-glutamine tracers perform poorly in this system in comparison to the optimal glucose tracers.

## Results and discussion

### Mammalian network model

**Figure 1:**
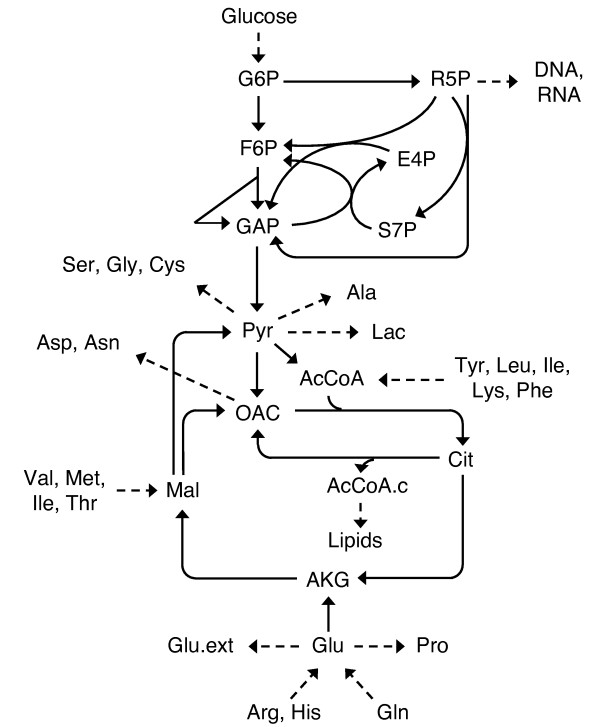
**Mammalian network model.** Network model for HEK-293 cells adapted from Henry et al. (2011). Two free fluxes exist in the model, the oxidative pentose phosphate pathway flux and pyruvate carboxylase flux. Dashed lines indicate measured external rates. Abbreviations: c = cytosol, ext = external.

The reaction network model of mammalian metabolism was adapted from Henry et al. [31] and is depicted in Figure 1 (see Additional file 1 for stoichiometry and atom transitions). External fluxes fixed by measurements are shown with dashed arrows. The model has two degrees of freedom, the oxidative pentose phosphate flux (oxPPP, G6P → R5P + CO_2_) and pyruvate carboxylase flux (PC, Pyr + CO_2_ → OAC). The lactate mass isotopomer distribution (MID) provides the additional constraints needed to determine the two free fluxes in the model. The Henry network model contains several substrates, including glucose and various amino acids. In this work, glucose and glutamine were considered the main carbon sources that could be ^13^C-labeled, while the remaining amino acids were treated as unlabeled.

### EMU basis vector decomposition of mammalian network model

**Figure 2:**
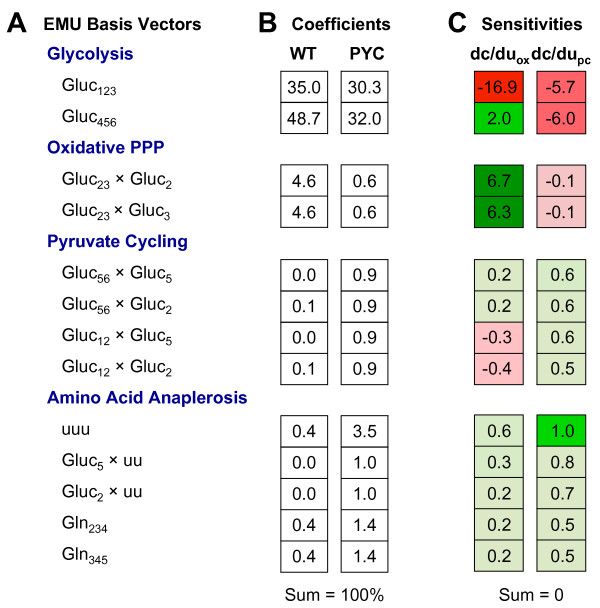
**EMU basis vectors, coefficients, and sensitivities.** Representative data for the EMU decomposition of the mammalian network model for lactate measurement. (A) Major EMU basis vectors from each of four metabolic pathways. (B) Contribution coefficients corresponding to EMU basis vectors for wild-type (WT) and PC-expressing (PYC) HEK-293 cells. (C) Sensitivities of coefficients to the free fluxes, oxPPP and PC (values are × 10^2^). Note: “u” refers to non-tracer (i.e. unlabeled) substrate atoms.

EMU decomposition of the mammalian network model in Figure 1 with lactate as the measured metabolite resulted in 156 EMU basis vectors (seeAdditional file 2). In other words, the system contains 156 possible ways of assembling the product lactate from the substrates. The EMU basis vectors with the largest fractional contributions, i.e. largest coefficients, and largest sensitivities of coefficients with respect to the two free fluxes in the model (oxPPP and PC fluxes), are shown in Figure 2A. For this system, the EMU basis vectors were mainly associated with one of four pathways: 1) glycolysis, 2) oxidative pentose phosphate pathway, 3) a pyruvate cycle that included anaplerosis by PC and cataplerosis by malic enzyme, and 4) a set of converging pathways that included anaplerosis from amino acids, e.g. glutamine, followed by cataplerosis by malic enzyme. The two characteristic EMU basis vectors corresponding to the formation of lactate through glycolysis were Gluc_123_ and Gluc_456_. Production of lactate through oxPPP also yielded two characteristic EMU basis vectors, Gluc_23_ × Gluc_2_ and Gluc_23_ × Gluc_3_; while synthesis of lactate via glutaminolysis produced, among others, the characteristic EMU basis vectors Gln_234_ and Gln_345_.

The EMU basis vector coefficients quantify the fractional contribution of each EMU basis vector’s labeling to the observed labeling of lactate (Figure 2)B. By definition, the coefficients must sum up to 100%. The coefficients shown in Figure 2B were calculated for two flux maps from the study by Henry et al. for two HEK-293 clones, wild-type cells (WT) and PC-expressing cells (PYC). The normalized flux values, relative to glucose uptake rate, are given in Table 1. WT cells converted most of glucose to lactate, at nearly half of the theoretical yield, and demonstrated moderate oxidative pentose phosphate flux (~30% of glucose influx), no PC activity, and low ME activity. The PC-expressing cells differed from WT cells by having lower lactate production, lower flux through oxPPP, increased TCA cycle flux, and increased anaplerosis and cataplerosis. For both HEK-293 clones, the largest contributions to lactate were from the EMU basis vectors Gluc_456_ and Gluc_123_, i.e. by glycolysis. In both cases, the contribution of Gluc_456_ was greater than that of Gluc_123_. This was due to the loss of C1 of glucose in oxPPP. The net lumped reaction of oxPPP is: 3 Gluc_123_ → Gluc_23_ × Gluc_2_ + Gluc_23_ × Gluc_3_ + 3 Gluc_1_ (CO_2_). Thus, an increase in oxPPP flux will increase the contributions of Gluc_23_ × Gluc_2_ and Gluc_23_ × Gluc_3_, slightly decrease Gluc_123_ (due to the loss of C1 carbons), and increase the contribution of Gluc_456_.

**Table 1:** Metabolic fluxes in the network model used for data simulation

**Flux**	**Wild type (WT)**	**PC cells (PYC)**
Gluc.ext → G6P	1.00	1.00
G6P → R5P + CO_2_	0.29	0.08
GAP → Pyr	1.88	1.93
Pyr → Lac	0.95	0.30
OAC + AcCoA → AKG + CO_2_	0.89	1.88
Pyr + CO_2_ → OAC	0.00	0.84
Mal → OAC	0.89	1.10
Mal → Pyr + CO_2_	0.15	1.11

Fluxes were taken from Henry et al. and were normalized to glucose uptake. Fluxes are shown for wild-type HEK-293 cells (WT) and HEK-293 cells expressing yeast pyruvate carboxylase (PYC).

Glycolysis and oxPPP accounted for ~93% of EMU basis vector contributions to lactate in WT cells. In contrast, due to a larger malic enzyme flux in PC-expressing cells, glycolysis and oxPPP contributed only ~63% to lactate in PYC cells. The remaining 37% of contributions resulted from anaplerosis. The two main anaplerotic reactions in this system were PC and glutaminolysis, and the largest single fractional contribution to lactate was from the EMU basic vector ‘uuu’ (u = unlabeled), that is, from EMU basis vector comprised of “non-tracer” substrates (~3.5%). The two dominant glutaminolysis contributions, Gln_234_ and Gln_345_, accounted for ~3% of the total contribution to lactate. The remaining contributions from glutamine EMUs were divided among 78 EMU basis vectors and contributed ~5% to lactate. The remaining ~25% of contributions were distributed among more than 40 EMU basis vectors, with no single contribution larger than 1.3%.

### EMU coefficient sensitivities and tracer experiment design strategy

The EMU basis vector coefficients provide valuable information regarding the dominant metabolic pathways in the system for a given set of fluxes. However, to address the tracer experiment design problem, i.e. how to select optimal tracers to accurately estimate fluxes in the model, additional information is needed. Specifically, the sensitivities of coefficients with respect to the free fluxes in the model provide useful additional data. Figure 2C shows the sensitivities calculated for the PYC flux map. The sensitivities quantify how the coefficients of EMU basis vectors are affected by changes in fluxes. A large sensitivity (either positive or negative) indicates that an EMU basis vector contribution changes significantly in response to a small change in a flux, and therefore, may be a good target for optimal tracer selection.

In this system, lactate mass isotopomers, i.e. M + 0, M + 1, M + 2, and M + 3, must provide the constraints needed to determine the two free fluxes in the model. Without MS fragmentation of lactate, at most three independent mass isotopomers can be obtained, i.e. the sum of lactate mass isotopomers must equal one. As previously demonstrated, the EMU basis vector formulation decouples isotopic labeling (i.e. the EMU basis vectors) from the free fluxes (i.e. the coefficients) in the system. Using this formulation, lactate mass isotopomers and sensitivities of lactate mass isotopomers with respect to the free fluxes can be expressed in a decoupled manner:

(1) LactÂ =Â BVÂ *Â c

(2) $$\text{d}\left(\text{Lact}\right)/\text{duÂ }=\text{Â BVÂ }*\text{Â dc}/\text{du}$$

In the above equations, the tracer labeling is confined to the EMU basis vectors matrix (BV), and the flux dependencies are given by the coefficient sensitivities (dc/du). In previous work, we demonstrated that the sum of sensitivities with respect to any flux in the system must sum up to zero [33]. Therefore, for each flux in the model there must be at least one positive sensitivity and at least one negative sensitivity, i.e. assuming not all sensitivities are zero. By selecting tracer substrates and substrate labeling judiciously, we can control how the EMU basis vector sensitivities will map onto lactate mass isotopomer sensitivities. Our strategy for optimal tracer experiment design is to choose the tracers such that the sensitivities of lactate mass isotopomers are maximized, and thus, we can obtain the best flux resolution. Our procedure for optimal tracer selection is therefore as follows:

1) Calculate EMU basis vector sensitivities for all free fluxes in the model

2) Identify the largest magnitude coefficient sensitivities for each free flux

3) Construct labeling rules to minimize the overlap of opposite-signed sensitivities (i.e. to prevent canceling out of sensitivities)

4) Construct labeling rules to maximize the overlap of same-signed sensitivities (i.e. to maximize the sensitivities of isotopomers)

5) List isotopic tracers that are consistent with the labeling rules and evaluate them by numerical simulations

In the next two sections we demonstrate how the above procedure can provide labeling rules to help in the selection of optimal tracer substrates and labeling patterns for the mammalian network model. By systematically applying the labeling rules we can drastically reduce the number of potential tracers to be evaluated by numerical simulations. Here, we demonstrate the application of this strategy to determine optimal tracers for elucidating the oxPPP and PC fluxes in the mammalian network model.

### Rational selection of tracers for estimating oxidative pentose phosphate pathway flux

**Figure 3:**
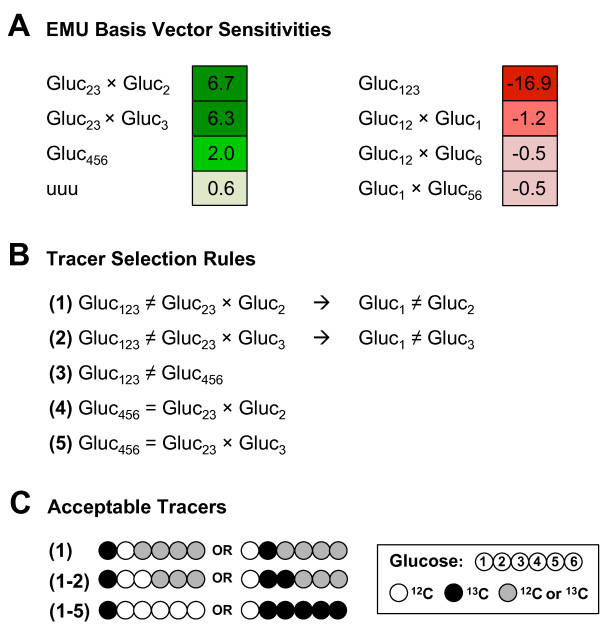
**Tracer selection for the oxidative pentose phosphate pathway flux.** (A) Positive and negative sensitivities for PYC cells. The dominant sensitivity is Gluc_123_ and the rules in (B) were derived to maximize this negative sensitivity. The rules are listed in sequential order of importance. (C) Schematic of acceptable tracers that coincide with the rules in (B). [2,3,4,5,6-^13^C]glucose was identified as the optimal tracer for the oxPPP flux.

The largest coefficient sensitivities for the oxPPP flux are shown in Figure 3A (full list in Additional file 2). The largest magnitude sensitivity was for the EMU basis vector Gluc_123_, which had a negative sensitivity value of −16.9. The negative value indicates that the contribution of Gluc_123_ sharply decreases for increasing oxPPP flux. The second largest negative sensitivity was >10-fold smaller in magnitude, Gluc_12_ × Gluc_1_ (−1.2). The largest positive sensitivities were for the EMU basis vectors Gluc_23_ × Gluc_2_ (+6.7), Gluc_23_ × Gluc_3_ (+6.3), and Gluc_456_ (+2.0).

To minimize the overlap of opposite-signed sensitivities (i.e. to prevent canceling out of sensitivities for lactate mass isotopomers), the EMU basis vectors with the positive sensitivities must not produce the same mass isotopomers as Gluc_123_. This criterion leads to a set of simple labeling rules that are shown in Figure 3B and depicted schematically in Figure 3C. First, Gluc_23_ × Gluc_2_ should differ from Gluc_123_. This can be achieved only if glucose carbons 1 and 2 are labeled differently. Similarly, Gluc_23_ × Gluc_3_ must be different from Gluc_123_, which requires that carbons 1 and 3 of glucose are labeled differently. For pure tracers, these two rules require that glucose carbons 2 and 3 are labeled in the same way (i.e. either both labeled or both unlabeled), hence combining the same-signed sensitivities, Gluc_23_ × Gluc_2_ and Gluc_23_ × Gluc_3_. Based on these two labeling rules it is clear that the first three carbon atoms of glucose must take the form of Gluc_100xxx_ or Gluc_011xxx_ (x = unspecified). The positive sensitivity value for Gluc_456_ sets the final constraints on the labeling of glucose. First, Gluc_456_ should differ from Gluc_123_; and second, Gluc_456_ should produce the same mass isotopomer as Gluc_23_ × Gluc_2_ and Gluc_23_ × Gluc_3_. With these rules, the list of 64 possible glucose tracers is narrowed down to only two glucose tracers, Gluc_100000_ (i.e. [1-^13^C]glucose) and Gluc_011111_ (i.e. [2,3,4,5,6-^13^C]glucose). The magnitude of the negative sensitivities for glucose tracers are shown in Additional file 3, Figure 1A. The tracers with the largest negative sensitivities all had the predicted labeling pattern of Gluc_100xxx_ or Gluc_011xxx_. The sensitivity analysis also revealed that glutamine tracers need not be considered as potential candidates for estimating oxPPP flux, since glutamine sensitivities were orders-of-magnitude smaller than glucose sensitivities for the oxPPP flux.

### Rational selection of tracers for estimating pyruvate carboxylase flux

**Figure 4:**
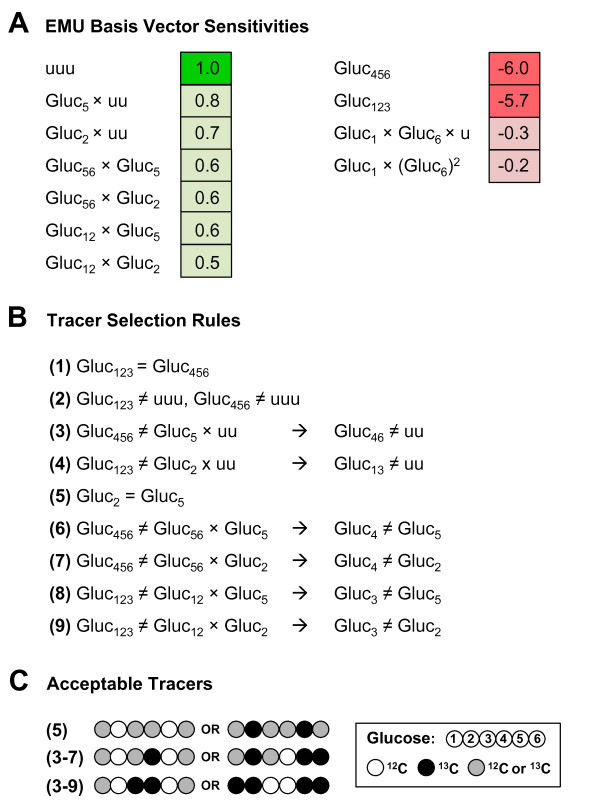
**Tracer selection for the pyruvate carboxylase flux.** (A) Positive and negative sensitivities for PYC cells. The dominant sensitivity is the sum of Gluc_123_ and Gluc_456_. (B) Rules were derived to prevent overlap of the positive sensitivities with Gluc_123_ and Gluc_456_. (C) Schematic of acceptable tracers that coincide with the rules in (B). [3,4-^13^C]glucose was determined as the optimal tracer for the PC flux.

The largest coefficient sensitivities for the PC flux are shown in Figure 4A (full list in Additional file 2). For the PC flux, the two largest magnitude sensitivities were for EMU basis vectors Gluc_123_ and Gluc_456_. Both sensitivities were negative (−6.0 and −5.7, respectively) and were much larger in magnitude than other negative sensitivities. The largest positive sensitivity (+1.0) was for the EMU basic vector ‘uuu’ (u = unlabeled), i.e. the EMU basis vector comprised of “non-tracer” substrates.

To maximize the magnitude of the negative sensitivities, Gluc_123_ and Gluc_456_ should produce the same mass isotopomer (i.e. Gluc_123_ = Gluc_456_), which is the first labeling rule for the PC flux shown in Figure 4B. The other rules are based on preventing the positive sensitivities from canceling out the two large negative sensitivities and efficiently grouping of the positive sensitivities. To prevent canceling out sensitivities, the EMU basis vector ‘uuu’ should be different from Gluc_123_ and Gluc_456_; in other words, at least one atom in Gluc_123_ must be labeled and at least one atom in Gluc_456_ must be labeled. Next, Gluc_5_ × uu and Gluc_2_ × uu should be different from Gluc_123_ and Gluc_456_, requiring that Gluc_13_ is labeled, and Gluc_46_ is labeled. These first four rules create constraints that all candidate tracers should obey.

Further investigation of the positive sensitivities highlights that several sensitivities can be grouped if Gluc_2_ = Gluc_5_ (e.g. Gluc_5_ × uu = Gluc_2_ × uu). The remaining rules, 6 – 9, in Figure 4B originate from preventing the overlap of the negative sensitivities of Gluc_123_ and Gluc_456_ with the remaining positive sensitivities (e.g. Gluc_56_ × Gluc_5_ must differ from Gluc_456_, which is only accomplished if Gluc_4_ is not the same as Gluc_5_). The tracer selection process is shown schematically in Figure 4C. With these rules, the list of 64 possible glucose tracers narrows down to only three potential glucose tracers, Gluc_001100_ (i.e. [3,4-^13^C]glucose), Gluc_110011_ (i.e. [1,2,5,6-^13^C]glucose), and Gluc_101101_ (i.e. [1,3,4,6-^13^C]glucose). The magnitude of the negative sensitivities for glucose tracers are shown in Additional file 3, Figure 1B. This analysis also reveals that glutamine tracers need not be considered as candidates for estimating the PC flux, since glutamine sensitivities were significantly smaller than glucose sensitivities. The two largest glutamine sensitivities were for Gln_234_ (+0.5) and Gln_345_ (+0.5).

### Comparison of tracers for mammalian network model

To demonstrate the effectiveness of our EMU methodology for tracer selection, we numerically simulated confidence intervals for all pure tracers, i.e. 64 glucose and 32 glutamine tracers. For each tracer, the lactate MID was calculated based on the PYC flux map, and then ^13^C-MFA was conducted to estimate fluxes and flux confidence intervals. The simulation results determined that the optimal tracer for the oxPPP flux was [2,3,4,5,6-^13^C]glucose and for the PC flux was [3,4-^13^C]glucose. Both of these tracers were predicted through the rational selection of tracers described in the two sections above. Confidence intervals for the optimal and other representative tracers are shown in Additional file 4. For the oxPPP flux, several other tracers had confidence intervals that approached but did not outperform [2,3,4,5,6-^13^C]glucose. As predicted by our methodology these tracers all corresponded to glucose labeling as Gluc_100xxx_ or Gluc_011xxx_. Unlike Gluc_011111_, these tracers violated rules 5 and 6 in Figure 3B, but their violation had minimal effect as the large negative sensitivity of Gluc_123_ was preserved. For the PC flux, [3,4-^13^C]glucose performed the best, and the second optimal tracer, [1,2,5,6-^13^C]glucose, was also predicted by our rational design criteria. These results correspond well with the observation regarding the positive sensitivities: there were >20 positive sensitivities, ranging from (+0.3 to +1.0), however, none of these sensitivities has Gluc_3_ or Gluc_4_ in the EMU basis vectors. As a result, the choice of Gluc_3_ and Gluc_4_ affected only the EMU basis vectors Gluc_123_ and Gluc_456_. If Gluc_3_ = Gluc_4_, the negative sensitivities can easily be segregated from the positive sensitivities. For example, if Gluc_3_ and Gluc_4_ are labeled, choosing the other glucose carbons to be unlabeled (Gluc_001100_) results in no overlap between the positive sensitivities and Gluc_123_/Gluc_456_. Also, if Gluc_3_ and Gluc_4_ are unlabeled, labeling the remaining glucose carbons (Gluc_110011_) results in minimal cancelling of sensitivities. Thus, it is not surprising that [3,4-^13^C]glucose and [1,2,5,6-^13^C]glucose were the two best tracers for PC flux resolution.

**Figure 5:**
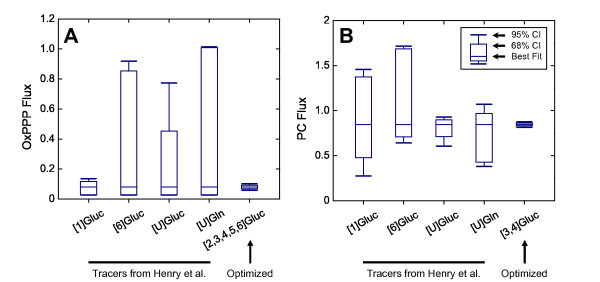
**Confidence intervals of fluxes from **^**13**^**C-MFA using simulated data.** The tracers used by Henry et al. (2011) were [1-^13^C], [6-^13^C], [U-^13^C]glucose, and [U-^13^C]glutamine. (A) Confidence intervals for the oxidative pentose phosphate flux (oxPPP). (B) Confidence intervals for the pyruvate carboxylase flux (PC). The tracers identified with our rational design criteria, [2,3,4,5,6-^13^C] and [3,4-^`13^C]glucose, outperformed the tracers selected by Henry et al.

To illustrate the improvement we obtained through rational tracer design, we compared the confidence intervals of our proposed tracers to those used in the study by Henry et al. [31]. Henry et al. used three glucose tracers and one glutamine tracer, [1-^13^C], [6-^13^C], and [U-^13^C]glucose, and [U-^13^C]glutamine. The tracers utilized by Henry et al., with the exception of [6-^13^C]glucose, are widely used for ^13^C-MFA in mammalian cells and are a good basis for comparison to our novel tracers. In this work, we identified more optimal tracers, namely [2,3,4,5,6-^13^C] for the oxPPP flux and [3,4-^13^C]glucose for the PC flux. Figure 5A displays the confidence intervals for the oxPPP flux for Henry’s tracers and our proposed tracer. For the oxPPP flux, [1-^13^C]glucose performed well, whereas both [6-^13^C] and [U-^13^C]glucose produced large confidence intervals. [U-^13^C]glutamine produced the largest confidence intervals, which is expected as no ^13^C-labeling information is present in the major EMU basis vectors. [2,3,4,5,6-^13^C]glucose produced the smallest confidence intervals of all the tracers, i.e. best flux resolution, displaying about 20-fold improvement of the confidence intervals over [6-^13^C]glucose, [U-^13^C]glucose, and [U-^13^C]glutamine. [2,3,4,5,6-^13^C]glucose also constituted a significant improvement (~2.5-fold) over [1-^13^C]glucose. Figure 5B illustrates the confidence intervals for the PC flux. [1-^13^C] and [6-^13^C]glucose performed poorly, while [U-^13^C]glucose was satisfactory. [U-^13^C]glutamine displayed intervals worse than [U-^13^C]glucose but better than both [1-^13^C] and [6-^13^C]glucose. [3,4-^13^C]glucose produced the smallest confidence interval, displaying a 5-fold improvement compared to [U-^13^C]glucose.

Finally, we evaluated the use of tracer mixtures. Mixtures of the optimal glucose tracers with unlabeled glucose resulted in larger confidence intervals of fluxes (see Additional file 5, Figure 3A & C). The optimal tracer, [2,3,4,5,6-^13^C]glucose, performed better than mixtures of [2,3-^13^C]glucose and [4,5,6-^13^C]glucose for oxPPP flux resolution; similarly [3,4-^13^C]glucose resulted in better PC confidence intervals than mixtures of [3-^13^C]glucose and [4-^13^C]glucose (see Additional file 5, Figure 3B & D). Overall, these results confirm that sensitivity-based criteria provide a rational approach for determining an appropriate design subspace for tracer selection that can result in drastic improvements in flux resolution.

## Conclusions

^13^C-Metabolic flux analysis has been increasingly used to observe *in vivo* fluxes in mammalian systems [34]. However, despite recent advances in both the experimental and computational aspects of ^13^C-MFA, little thought is often given to which tracers should be selected for a given network, and perhaps more importantly why these tracers are optimal.

In this contribution, we provide a new perspective for rational-based selection of ^13^C-tracers. Our methodology is based on the previously described concept of EMU basis vectors [33]. The EMU basis vector methodology is a very attractive strategy for investigating tracer selection as the tracer labeling is decoupled from the flux dependencies. In contrast to simulation-based optimal design [17,30,35-38], we focused on rational grouping of the flux-dependent coefficient sensitivities such that we could maximize the sensitivity of a single isotopomer for each free flux. For each free flux, we sorted the coefficient sensitivities by sign and decreasing magnitude. We then identified the largest magnitude(s) sensitivities. In the case of multiple moderate to large sensitivities, we collapsed those of the same sign onto a single isotopomer, while keeping those of the opposite sign on different isotopomers. Subsequently, we attempted to further group same-signed sensitivities, with emphasis on maximizing the largest sensitivity. In this process, we created labeling rules, which set constraints on the basis vector matrix and hence the possible substrate labeling schemes. Using this rationale, we obtained a significant reduction in the number of tracers, from 96 in total (64 glucose and 32 glutamine) to a handful of possible candidates. We predicted two novel optimal tracers, which were not previously considered for mammalian systems. For the oxPPP flux we determined that [2,3,4,5,6-^13^C]glucose would be the best tracer and [3,4-^13^C]glucose would be optimal for the PC flux. When we compared these *a priori* selections to simulation experiments from Henry’s PYC flux map, we observed drastic improvement in flux resolution for both the oxPPP and PC fluxes.

One practical insight this contribution provides is in the identification of feasible substrates for ^13^C-tracers through coefficient sensitivities. For a defined network, a flux map, and a measurement set, the dc/du values are fixed (see Eq. 2). Regardless of tracer choice and the respective labeling pattern, the coefficient sensitivities will not be affected. The method proposed in this work relies on rational grouping of the coefficient sensitivities to maximize the sensitivity of a single isotopomer for each free flux. In order to do this, it is important to choose a substrate that has large dc/du values corresponding with its respective EMU basis vectors. In essence, the coefficient sensitivities can be viewed as the *potential* to obtaining a large measurement sensitivity. Substrates with a larger potential are inherently better suited as tracer candidates for ^13^C-MFA.

To illustrate this concept, two possible substrates were considered in this work, glucose and glutamine. Glucose EMU basis vectors had large magnitude sensitivities for both the oxPPP flux (Gluc_123_, -16.9) and the PC flux (Gluc_123_, -5.7; Gluc_456_, -6.0); in contrast, the dominant glutamine EMU basis vectors had small sensitivities for the oxPPP flux (Gln_234_ = Gln_345_, +0.2) and the PC flux (Gln_234_ = Gln_345_, +0.5). As glutamine sensitivities were an order of magnitude smaller than glucose sensitivities, glutamine was clearly not an optimal tracer for this network with lactate as the measured metabolite. The simulation results validated this assessment, as [U-^13^C]glutamine was shown to be a poor tracer for elucidation of both the oxPPP and PC fluxes.

Intrinsically, the poor resolution of the oxPPP flux, when assessed with glutamine tracers, is reasonable as no labeling from glutamine can enter into oxPPP. More surprising is the poor resolution of the PC flux. One explanation for the poor PC flux resolution is the distance of the measurement, i.e. lactate, from glutamine and the resulting dilutions that occur at metabolites α-ketoglutarate and pyruvate. To remedy this issue, a TCA cycle intermediate (oxaloacetate or α-ketoglutarate) could be used as additional measurement; however, even if a TCA cycle intermediate is used, the measurement remains insensitive to the PC flux for [U-^13^C]glutamine (results not shown). We have demonstrated in this contribution that given a measurement, we can determine logical tracers for elucidation of a flux of interest; however, the converse, given a tracer, which measurements should be chosen to determine the flux is not trivial, or well understood. An understanding of both relationships will be crucial to designing optimal ^13^C-tracer experiments.

This work also demonstrates why it is often difficult to resolve all fluxes in a network with high confidence. In this network model, ^13^C-labeling rules for resolving the oxPPP flux were inherently contradicting the rules for optimally resolving the PC flux. Thus, by trying to resolve one flux better, the resolution of the other flux worsened. For example, in this work, to resolve the oxPPP flux it was desirable to have Gluc_123_ ≠ Gluc_456_; however for the PC flux, it was pressing to have Gluc_123_ = Gluc_456_. Both of these rules cannot be satisfied in a single tracer experiment. To select a single tracer to resolve both fluxes, the coefficient sensitivities and their magnitudes must be considered for both of the free fluxes. The most important criterion for oxPPP was that the strongly negative Gluc_123_ sensitivity collapsed on a different isotopomer than Gluc_23_ × Gluc_2_ and Gluc_23_ × Gluc_3_. Crucial for PC flux resolution was that Gluc_123_ and Gluc_456_ produced the same isotopomer. These two constraints can be satisfied together, if the stipulation for the oxPPP rule set is relaxed, such that Gluc_456_ can differ from Gluc_123_. Since the Gluc_456_ sensitivity is only about 2% and Gluc_123_ is almost −17%, this is a reasonable compromise. With the adapted rules, the first three carbons of glucose must be either [100] or [011] labeled, with the last three carbons being M + 1 or M + 2 labeled, respectively. Through careful selection of which tracer rules to violate, ideally ones that have a lesser impact on the maximum sensitivity for a given flux, a single tracer can be chosen to resolve both free fluxes with precision that approaches that of the optimal tracers we suggested (see Additional file 6). Another important observation regarding flux resolution corresponds to the range of the sensitivity values. In the case of the oxPPP flux, there are three dominant sensitivities (Gluc_123_, Gluc_23_ × Gluc_2_, and Gluc_23_ × Gluc_3_). Violation of rules involving combinations of these three sensitivities, has drastic effect on the resulting confidence intervals. However, in the case where many sensitivities are of similar magnitude (e.g. the positive sensitivities for PC flux), violation of individual rules (5–9 in Figure 4) can have less severe consequences. For example, [2,3,4,6-^13^C]glucose in Additional file 6 violates rules 5, 7, and 9, but retains confidence intervals about twice as large as those of [3,4-^13^C]glucose.

In simple cases, such as this network, a single tracer and a single measurement may be capable of resolving all free fluxes with high fidelity; however, as the number of free fluxes increases in a network, not all sensitivity rules for each flux can be satisfied, resulting in smaller magnitudes of isotopomer sensitivity and loss of confidence in the estimated flux values. This raises an important question of how to minimize the effects of conflicting sensitivity rules, and hence improve confidence intervals. There are two feasible approaches to address this issue. The first option involves a single-tracer design with the addition of more independent measurements. The additional isotopomers may allow more flexibility when satisfying the sensitivity criteria. One concern, however, is that contradictory rules may still exist and result in poor flux resolution. In this case, additional measurements may have only marginal effect on flux resolution [35], thus requiring another approach to achieve better flux results. A second alternative to improve flux resolution is to conduct parallel labeling experiments, where specific tracers are designed to be optimal for specific fluxes in the model. By integrating labeling data from such parallel labeling experiments, fluxes can be resolved at a high resolution that can never be achieved using any single tracer experiment. The obvious drawback to this method is tracer availability and cost, and the requirement of good biological reproducibility. The tracer selection methodology presented here gives clear insight into why flux resolution is challenging and highlights the need for investigation of not just tracer and measurement choice, but also the manner in which tracer experiments are conducted.

This work also offers some experimental insights regarding the usage of [1-^13^C]glucose for oxPPP resolution. The results shown here demonstrate that [2,3,4,5,6-^13^C]glucose is a more effective tracer. To further expand on this point, we numerically simulated oxPPP confidence interval for [1-^13^C]glucose and [2,3,4,5,6-^13^C]glucose. A grid search for the two free fluxes (oxPPP and PC) was conducted to evaluate the effect of the free fluxes on the resulting confidence intervals. Overall, for this network with lactate as the measurement, [2,3,4,5,6-^13^C]glucose performed as well as, and in the majority of cases, better than [1-^13^C]glucose across the entire flux space (see Additional file 7).

Another insight this work provides is into experiment design with mixtures of ^13^C-tracers. Often times, especially in mammalian cell culture, there will be unlabeled glucose and amino acids in the media. As shown in Additional file 5, the addition of unlabeled glucose adversely affects the flux confidence intervals for the optimal tracers. This can be explained through the EMU basis vector sensitivities. For example, consider the sensitivity of Gluc_123_ for the oxPPP flux. When pure [2,3,4,5,6-^13^C]glucose is used, the full sensitivity of Gluc_123_ (−16.9) contributes to the M + 2 isotopomer; however for a 50/50 mixture of [2,3,4,5,6-^13^C]glucose and unlabeled glucose, only half of the Gluc_123_ sensitivity (−8.5) contributes to M + 2 and the other half contributes to M + 0. Unlabeled glucose in this example results in a decrease in the maximum sensitivity obtainable. As a result, the flux observability suffers. Similarly, with mixtures of [2,3-^13^C]glucose and [4,5,6-^13^C]glucose, the maximum obtainable sensitivity was decreased, also resulting in poorer confidence intervals.

Lastly, it is important to discuss the limitations of the Henry model and how it pertains to the proposed methodology. The Henry model did not include commonly accepted reaction reversibilities, such as transketolase (TK) and transaldolase (TA) in the pentose phosphate pathway as well as malate dehydrogenase (MDH). Reversibility of TK and TA will allow back-mixing of labeling in the pentose phosphate pathway and the reversibility of MDH will result in additional pyruvate cycling via PC, MDH, and malic enzyme acting in tandem (i.e. pyruvate → oxaloacetate → malate → pyruvate). In general, inclusion of reversible reactions may or may not increase the number of EMU basis vectors depending on whether the reversible reactions create new, independent “EMU pathways”. The fractional contributions will change, as the coefficients will be functions of additional free fluxes. The most notable change will be in the coefficient sensitivities. In addition to sensitivities with respect to the oxPPP and PC flux, each coefficient will have a sensitivity to each reversible flux. The tracer selection process based on our methodology remains the same; however, it may not be feasible to resolve all fluxes with the given measurement(s). For example, in the system described here, lactate only has three independent mass isotopomers, i.e. assuming the complete lactate molecule is measured and no other MS fragments of lactate are available. With the addition of TK, TA, and MDH reversibilities, there will be six free fluxes, and thus it will not be possible to resolve all these fluxes with lactate as the only measurement. To demonstrate this, oxPPP and PC confidence intervals were simulated for various glucose tracers, where the network model included TK, TA, and MDH. The results are shown in Additional file 8. The uncertainty due to the inability to resolve all six free fluxes caused broadening of the confidence intervals. The best-performing tracers for the oxPPP and PC flux, however, remained the same.

In addition to reversible reactions, compartmentation was also neglected in the Henry model, meaning that parallel reactions in the cytosol and mitochondria were not distinguished in this model. Experimentally, measuring fluxes in separate compartments is difficult without isolation of metabolites located in the different cellular compartments [39]. As advances are made to overcome this technical challenge, the methodology we have presented here will still be applicable, as the rational steps proposed are independent of the model. Regardless of the number of free fluxes, sensitivity criteria can be applied to evaluate principles for each free flux. As the model complexity increases, however, more measurements or parallel experiments may be necessary as discussed above.

In summary, the results in this paper demonstrate that optimal tracer experiment design does not need to be a pure simulation-based trial-and-error process. But rather, rational insights into tracer design can be gained through application of the EMU basis vector methodology. Through careful analysis of sensitivities, with focus on maximizing isotopomer sensitivity, labeling rules can be constructed, which guide the selection of ^13^C-tracers for a given network. Depending on the size and complexity of the network, the proposed methodology may provide a single optimal tracer, as in [3,4-^13^C]glucose for the PC flux; or perhaps more likely, the method will provide a reduced list of feasible tracers. This reduction of plausible tracer schemes, whether complete or partial, can significantly ease the computational burden for further tracer experiment design optimization. Going forward, further emphasis should be placed on understanding the interdependencies between measurements in conjunction with a rational selection of tracers and the overarching philosophy of isotopic experiment design. One important issue to address is whether a tracer experiment should be completed in isolation, i.e. one tracer experiment to elucidate all the fluxes, or whether parallel experiments are better suited, i.e. several tracer experiments with each resolving a different subset of the fluxes. Ultimately, further investigation of the correlations between flux resolution, the measurement set, and the ^13^C-tracer must be conducted. A deeper understanding of these relationships will allow for more powerful isotopic experiment design for ^13^C-MFA.

## Methods

### Nomenclature

The tracer experiment design framework presented here is built using mass isotopomer distributions (MIDs) of EMUs as state variables [13]. An EMU is defined as a specific subset of metabolite’s atoms. We use a subscript notation to denote atoms present in an EMU. For example, A_234_ indicates that the EMU is comprised of atoms 2, 3, and 4 of metabolite A. Furthermore, a subscript notation (with ones and zeros) is used to denote the labeling patterns of isotopomers. For example, A_1100_ indicates that metabolite A has four atoms and that atoms 1 and 2 are labeled and atoms 3 and 4 are unlabeled.

An MID is a vector that contains the fractional abundances of each mass isotopomer of an EMU, i.e. [M + 0, M + 1, …, M + *n*] for an EMU of size *n*. A convolution (or Cauchy product) describes the condensation of two EMU’s and is denoted by “×.” For example, if C_123_ = A_12_ × B_1_, then the MID of C_123_ will be expressed as:

(3) $$C_{123,M+0}=A_{12,M+0}\;B_{1,M+0}$$

(4) $$C_{123,M+1}=A_{12,M+1}\;B_{1,M+0}+A_{12,M+0}\;B_{1,M+1}$$

(5) $$C_{123,M+2}=A_{12,M+2}\;B_{1,M+0}+A_{12,M+1}\;B_{1,M+1}$$

(6) $$C_{123,M+3}=A_{12,M+2}\;B_{1,M+1}$$

In this study, we only consider pure tracers (i.e. not mixtures of tracers), which means that the MID of an EMU such as Gluc_123_ is equivalent to any convolution of EMUs involving the same atoms, e.g. Gluc_123_ will produce same MID as Gluc_12_ × Gluc_3_.

An EMU basis vector is a unique way for assembling substrate EMUs to form the measured metabolite. The MID of the measured metabolite is a linear combination of EMU basis vector MIDs. The coefficients are solely a function of free fluxes and quantify the “weights” of each EMU basis vector to the measurement [33].

### Network model

The reaction network model of mammalian metabolism by Henry et al. [31] consists of central carbon metabolic pathways, including glycolysis, pentose phosphate pathway, tricarboxylic acid cycle, anaplerotic and cataplerotic reactions, as well as metabolism of amino acids. Reversible reactions and intracellular compartmentation were not taken into account in this model; however, scrambling of ^13^C-labeling due to rotational symmetry of fumarate and succinate was considered. In total, the model contains 29 reactions and 29 metabolites, with 15 balanced intracellular metabolites and 13 measured extracellular metabolites (see Additional file 1). The thirteen fluxes fixed by the external measurements are shown with dashed arrows in Figure 1. The model has two degrees of freedom, the oxidative pentose phosphate flux (oxPPP, G6P → R5P + CO_2_) and pyruvate carboxylase flux (PC, Pyr + CO_2_ → OAC). Lactate mass isotopomers provide the additional constraints needed to determine the two free fluxes in the model. The Henry network model contains several substrates, including glucose and various amino acids. In this work, glucose and glutamine were considered the main carbon sources that could be ^13^C-labeled, while the remaining amino acids were treated as unlabeled. The identities of unlabeled amino acid substrates were collectively referred to as “non-tracer” substrates in the EMU decomposition. The two flux maps estimated by Henry et al. for HEK-293 cells (WT) and PC-expressing HEK-293 cells (PYC) were used as reference in this study. The PYC flux map was used for simulations and for optimal tracer experiment design.

### EMU decomposition

EMU decomposition of the metabolic network model was accomplished using Metran software [22]. The resulting EMU networks were decoupled into separate and smaller subnetworks using the technique described by Young et al. [40], and simplified using the technique described by Antoniewicz et al. [13]. The EMU basis vectors for extracellular lactate were enumerated using the technique by Crown and Antoniewicz [33]. In the context of this work, an EMU basis vector refers to a unique way of assembling substrate EMUs to form the secreted product lactate. The MID of lactate can be interpreted as a linear combination of EMU basis vector MIDs. The EMU basis vector coefficients quantify the fractional contribution of each EMU basis vector to the observed labeling of lactate. Coefficient sensitivities were calculated using finite differences as before [33].

### Metabolic flux analysis

^13^C-MFA was performed using Metran software, which is built on the EMU framework. In short, fluxes were estimated by minimizing the variance-weighted sum of squared residuals between the simulated and model-predicted MIDs using least-squares regression [41]. In all cases, flux estimation was repeated at least ten times starting with random initial values for all fluxes to find a global solution. At convergence, standard deviations, and 68% and 95% confidence intervals for all fluxes were calculated using the parameter continuation technique [41]. The technique is based on evaluating the profile of SSR as a function of one flux, while the values for the remaining fluxes are optimized. The 68% and 95% confidence intervals of an evaluated flux correspond to flux values that increased SSR by less than 1.00 and 3.84, respectively [41].

## Abbreviations

BV: EMU basis vectors; ^13^C-MFA: ^13^C-metabolic flux analysis; EMU: Elementary metabolite unit; Gluc: Glucose; Gln: Glutamine; MDH: Malate dehydrogenase; MID: Mass isotopomer distribution; MS: Mass spectrometry; oxPPP: Oxidative pentose phosphate pathway; PC: Pyruvate carboxylase; PYC: PC-expressing cells; TA: Transaldolase; TK: Transketolase; WT: Wild-type cells.

## Competing interests

The authors declare that they have no competing interests.

## Authors' contributions

MRA, SBC, and WSA conceived the idea for this study. SBC conducted the simulations, performed data analysis, and drafted the manuscript. MRA and WSA provided support with data analysis and finalizing the manuscript. All authors read and approved the final manuscript.

## Supplementary Material

Additional file 1 **– Stoichiometry, carbon transitions, and assumed fluxes for network model**. Title: Stoichiometry, carbon transitions, and assumed fluxes for network model. Description: Necessary information to reproduce simulation results in this manuscript. Click here for file

Additional file 2 **– EMU basis vectors, coefficients, and sensitivities**. Title: EMU basis vectors, coefficients, and sensitivities. Description: Exhaustive listing of EMU basis vectors, coefficients, and sensitivities. Click here for file

Additional file 3 **– Sensitivities of tracers to the oxPPP and PC fluxes**. Title: Sensitivities of tracers to the oxPPP and PC fluxes. Description: Maximum sensitivity for glucose tracers with respect to (A) oxPPP flux and (B) PC flux. Click here for file

Additional file 4 **– Confidence intervals of oxPPP and PC fluxes for various glucose tracers**. Title: Confidence intervals of oxPPP and PC fluxes for various glucose tracers. Description: Representative confidence intervals for glucose tracers for (A) oxPPP flux and (B) PC flux. Click here for file

Additional file 5 **– Effect of mixtures on oxPPP and PC confidence intervals**. Title: Effect of mixtures on oxPPP and PC confidence intervals. Description: Mixture effects of unlabeled glucose tracer for optimal oxPPP and PC tracers (A & C). Comparison of commercially available tracers mixtures versus the custom synthesized counterparts for oxPPP and PC fluxes (B & D). Click here for file

Additional file 6 **– Resolution of oxPPP and PC fluxes with a single tracer**. Title: Resolution of oxPPP and PC fluxes with a single tracer. Description: Comparison of confidence intervals for (A) oxPPP flux and (B) PC flux for optimal tracers ([1-^13^C]glucose, [2,3,4,5,6-^13^C]glucose, and [3,4-^13^C]glucose), and others. Click here for file

Additional file 7 **– Comparison of [1-**^**13**^**C]glucose and [2,3,4,5,6-**^**13**^**C]glucose for oxPPP resolution**. Title: Comparison of [1-^13^C]glucose and [2,3,4,5,6-^13^C]glucose for oxPPP resolution. Description: Range of confidence intervals for [1-^13^C]glucose and [2,3,4,5,6-^13^C]glucose over various combinations of oxPPP and PC flux values. Click here for file

Additional file 8 **– Comparison of reaction reversibilities on confidence intervals for oxPPP and PC fluxes**. Title: Comparison of reaction reversibilities on confidence intervals for oxPPP and PC fluxes. Description: Simulation of (A) oxPPP and (B) PC confidence intervals for network model without reversible fluxes (blue bars) and with reversible reactions included (red bars). For simulations including reversible reactions, the assumed exchange fluxes and normalized flux values were: transketolase (0.08), transaldolase (0.08), and malate dehydrogenase (1.1). Click here for file
