# Supplementary material for: Rational design of 13C-labeling experiments for metabolic flux analysis in mammalian cells
Source: BMC Syst Biol. 2012 May 16;6:43. doi: 10.1186/1752-0509-6-43 (PMC3490712; doi:10.1186/1752-0509-6-43)
Supplement: Additional file 1 — – Stoichiometry, carbon transitions, and assumed fluxes for network model. Title: Stoichiometry, carbon transitions, and assumed fluxes for network model. Description: Necessary information to reproduce simulation results in this manuscript. [file 1752-0509-6-43-S1.pdf]

Supp. Table 1 – Stochiometry, carbon transitions, and assumed fluxes for network model

No.	Reaction	Atom transitions	PYC flux*
1	Glc.ext → G6P	abcdef → abcdef	1.75
2	G6P → F6P	abcdef → abcdef	1.61
3	G6P → R5P + CO_2	abcdef → bcdef + a	0.14
4	R5P + R5P → S7P + G3P	abcde +fghij → abfghij + cde	0.03
5	R5P + E4P → F6P + G3P	abcde +fghi → abfghi + cde	0.03
6	S7P + G3P → F6P + E4P	abcdefg → abchij + defg	0.03
7	F6P → G3P + G3P	abcdef → cba + def	1.67
8	G3P → Pyr	abc → abc	3.37
9	Pyr → Lac	abc → abc	0.53
10	Pyr → AcCoA + CO_2	abc → bc + a	3.17
11	Pyr + CO_2 → Oac	abc + d → abcd	1.47
12	AcCoA + Oac → Cit	ab + cdef → fedbac	3.89
13	Cit → AKG + CO_2	abcdef → abcde + f	3.29
14	AKG → Mal + CO_2	abcde → ½ bcde + ½ edcb + a	3.63
15	Mal → Oac	abcd -> abcd	1.92
16	Mal → Pyr + CO_2	abcd -> abc + d	1.95
17	Pyr + Glu → Ala + AKG	abc + defgh → abc + defgh	0.05
18	Glu → AKG	abcde → abcde	0.30
19	Cit → Lipids + Oac	abcdef + fcba	0.60
20	AA's (Tyr, Leu, Ile, Lys, Phe) → AcCoA	ab → ab	0.72
21	AA's (Val, Met, Ile, Thr) → Mal	abcd → abcd	0.23
22	Pyr → AA's (Ser, Gly, Cys)	abc → abc	0.10
23	OAC → AA's (Asp, Asn)	abcd → abcd	0.10
24	AA's (Arg, His) → Glu	abcde → abcde	0.07
25	Gln → Glu	abcde → abcde	0.55
26	Glu → Pro	abcde → abcde	0.15
27	Glu → Glu.ext	abcde → abcde	0.13
28	R5P → DNA/RNA	abcde → abcde	0.05
29	CO_2 → CO_2.ext	a → a	10.7

* Flux values adapted from Henry et al. In the main paper, all flux values were normalized to glucose uptake rate.
