# Supplementary material for: Rational design of 13C-labeling experiments for metabolic flux analysis in mammalian cells
Source: BMC Syst Biol. 2012 May 16;6:43. doi: 10.1186/1752-0509-6-43 (PMC3490712; doi:10.1186/1752-0509-6-43)
Supplement: Additional file 2 — – EMU basis vectors, coefficients, and sensitivities. Title: EMU basis vectors, coefficients, and sensitivities. Description: Exhaustive listing of EMU basis vectors, coefficients, and sensitivities. [file 1752-0509-6-43-S2.pdf]

Supp. Table 2 - EMU basis vectors, coefficients, and sensitivities

**Coefficients**	**Positive Sens.**	**Neg Sens.**
< 1%	< 1%	> -1%
1 to 5%	1 to 5%	-5 to -1%
> 5%	> 5%	< -5%

g = glucose
n = glutamine
x = non-tracer substrate

Coefficients

Basis vectors	"Wild Type"	PYC-express.
'g123'	35.0%	30.3%
'g12g2'	0.1%	0.9%
'g12g3'	0.0%	0.0%
'g12g5'	0.0%	0.9%
'g12g6'	0.3%	1.2%
'g12n3'	0.1%	0.2%
'g12n4'	0.1%	0.2%
'g12x'	0.1%	1.3%
'g1g12'	0.2%	1.1%
'g1g1g1'	0.1%	0.1%
'g1g1g2'	0.1%	0.3%
'g1g1g3'	0.0%	0.0%
'g1g1g5'	0.0%	0.3%
'g1g1g6'	0.3%	0.4%
'g1g1n3'	0.0%	0.1%
'g1g1n4'	0.0%	0.1%
'g1g1x'	0.1%	0.5%
'g1g23'	0.1%	0.0%
'g1g2g2'	0.0%	0.1%
'g1g2g3'	0.0%	0.0%
'g1g2g5'	0.0%	0.2%
'g1g2g6'	0.2%	0.6%
'g1g2n3'	0.0%	0.1%
'g1g2n4'	0.0%	0.1%
'g1g2x'	0.0%	0.6%
'g1g3g3'	0.0%	0.0%
'g1g3g5'	0.0%	0.0%
'g1g3g6'	0.0%	0.0%
'g1g3n3'	0.0%	0.0%
'g1g3n4'	0.0%	0.0%
'g1g3x'	0.0%	0.0%
'g1g56'	0.3%	1.2%
'g1g5g5'	0.0%	0.1%
'g1g5g6'	0.0%	0.6%
'g1g5n3'	0.0%	0.1%
'g1g5n4'	0.0%	0.1%
'g1g5x'	0.0%	0.6%
'g1g6g6'	0.4%	0.5%
'g1g6n3'	0.1%	0.2%
'g1g6n4'	0.1%	0.2%
'g1g6x'	0.3%	1.0%
'g1n34'	0.1%	0.3%
'g1n3n3'	0.0%	0.0%
'g1n3n4'	0.0%	0.0%
'g1n3x'	0.0%	0.2%
'g1n4n4'	0.0%	0.0%
'g1n4x'	0.0%	0.2%
'g1xx'	0.2%	1.1%
'g23g3'	4.6%	0.6%
'g23g5'	0.0%	0.0%
'g23g6'	0.1%	0.0%
'g23n3'	0.0%	0.0%
'g23n4'	0.0%	0.0%
'g23x'	0.0%	0.0%
'g2g23'	4.6%	0.6%
'g2g2g2'	0.0%	0.0%
'g2g2g3'	0.0%	0.0%
'g2g2g5'	0.0%	0.0%
'g2g2g6'	0.0%	0.1%
'g2g2n3'	0.0%	0.0%
'g2g2n4'	0.0%	0.0%
'g2g2x'	0.0%	0.1%
'g2g3g3'	0.0%	0.0%
'g2g3g5'	0.0%	0.0%
'g2g3g6'	0.0%	0.0%
'g2g3n3'	0.0%	0.0%
'g2g3n4'	0.0%	0.0%
'g2g3x'	0.0%	0.0%
'g2g56'	0.1%	0.9%
'g2g5g5'	0.0%	0.0%
'g2g5g6'	0.0%	0.2%
'g2g5n3'	0.0%	0.0%
'g2g5n4'	0.0%	0.0%
'g2g5x'	0.0%	0.2%
'g2g6g6'	0.1%	0.3%

Sensitivities for PYC cells

Basis vectors	oxPPP	PC
'g123'	-16.9%	-5.7%
'g12g2'	-0.4%	0.5%
'g12g3'	0.3%	0.0%
'g12g5'	-0.3%	0.6%
'g12g6'	-0.5%	0.3%
'g12n3'	-0.1%	0.0%
'g12n4'	-0.1%	0.0%
'g12x'	-0.5%	0.4%
'g1g12'	-1.2%	0.3%
'g1g1g1'	-0.2%	-0.1%
'g1g1g2'	-0.3%	0.1%
'g1g1g3'	0.1%	0.0%
'g1g1g5'	-0.3%	0.1%
'g1g1g6'	-0.5%	-0.2%
'g1g1n3'	-0.1%	0.0%
'g1g1n4'	-0.1%	0.0%
'g1g1x'	-0.5%	-0.1%
'g1g23'	0.5%	0.0%
'g1g2g2'	0.1%	0.1%
'g1g2g3'	0.1%	0.0%
'g1g2g5'	0.1%	0.3%
'g1g2g6'	-0.2%	0.2%
'g1g2n3'	0.0%	0.0%
'g1g2n4'	0.0%	0.0%
'g1g2x'	-0.1%	0.3%
'g1g3g3'	0.0%	0.0%
'g1g3g5'	0.1%	0.0%
'g1g3g6'	0.2%	0.0%
'g1g3n3'	0.0%	0.0%
'g1g3n4'	0.0%	0.0%
'g1g3x'	0.2%	0.0%
'g1g56'	-0.5%	0.3%
'g1g5g5'	0.0%	0.1%
'g1g5g6'	-0.2%	0.2%
'g1g5n3'	0.0%	0.0%
'g1g5n4'	0.0%	0.0%
'g1g5x'	-0.2%	0.3%
'g1g6g6'	-0.2%	-0.2%
'g1g6n3'	-0.1%	0.0%
'g1g6n4'	-0.1%	0.0%
'g1g6x'	-0.5%	-0.3%
'g1n34'	-0.1%	0.0%
'g1n3n3'	0.0%	0.0%
'g1n3n4'	0.0%	0.0%
'g1n3x'	-0.1%	0.0%
'g1n4n4'	0.0%	0.0%
'g1n4x'	-0.1%	0.0%
'g1xx'	-0.5%	-0.1%
'g23g3'	6.3%	-0.1%
'g23g5'	0.4%	0.0%
'g23g6'	0.5%	0.0%
'g23n3'	0.1%	0.0%
'g23n4'	0.1%	0.0%
'g23x'	0.5%	0.0%
'g2g23'	6.7%	-0.1%
'g2g2g2'	0.0%	0.0%
'g2g2g3'	0.0%	0.0%
'g2g2g5'	0.1%	0.1%
'g2g2g6'	0.2%	0.1%
'g2g2n3'	0.0%	0.0%
'g2g2n4'	0.0%	0.0%
'g2g2x'	0.2%	0.1%
'g2g3g3'	0.0%	0.0%
'g2g3g5'	0.0%	0.0%
'g2g3g6'	0.1%	0.0%
'g2g3n3'	0.0%	0.0%
'g2g3n4'	0.0%	0.0%
'g2g3x'	0.1%	0.0%
'g2g56'	0.2%	0.6%
'g2g5g5'	0.0%	0.1%
'g2g5g6'	0.2%	0.3%
'g2g5n3'	0.0%	0.0%
'g2g5n4'	0.0%	0.0%
'g2g5x'	0.2%	0.3%
'g2g6g6'	0.1%	0.1%

'g2g6n3'	0.0%	0.1%
'g2g6n4'	0.0%	0.1%
'g2g6x'	0.1%	0.6%
'g2n34'	0.0%	0.1%
'g2n3n3'	0.0%	0.0%
'g2n3n4'	0.0%	0.0%
'g2n3x'	0.0%	0.1%
'g2n4n4'	0.0%	0.0%
'g2n4x'	0.0%	0.1%
'g2xx'	0.0%	1.0%
'g3g3g3'	0.0%	0.0%
'g3g3g5'	0.0%	0.0%
'g3g3g6'	0.0%	0.0%
'g3g3n3'	0.0%	0.0%
'g3g3n4'	0.0%	0.0%
'g3g3x'	0.0%	0.0%
'g3g56'	0.0%	0.0%
'g3g5g5'	0.0%	0.0%
'g3g5g6'	0.0%	0.0%
'g3g5n3'	0.0%	0.0%
'g3g5n4'	0.0%	0.0%
'g3g5x'	0.0%	0.0%
'g3g6g6'	0.0%	0.0%
'g3g6n3'	0.0%	0.0%
'g3g6n4'	0.0%	0.0%
'g3g6x'	0.0%	0.0%
'g3n34'	0.0%	0.0%
'g3n3n3'	0.0%	0.0%
'g3n3n4'	0.0%	0.0%
'g3n3x'	0.0%	0.0%
'g3n4n4'	0.0%	0.0%
'g3n4x'	0.0%	0.0%
'g3xx'	0.0%	0.0%
'g456'	48.7%	32.0%
'g56g6'	0.5%	1.3%
'g56n3'	0.1%	0.2%
'g56n4'	0.1%	0.2%
'g56x'	0.2%	1.3%
'g5g56'	0.0%	0.9%
'g5g5g5'	0.0%	0.0%
'g5g5g6'	0.0%	0.1%
'g5g5n3'	0.0%	0.0%
'g5g5n4'	0.0%	0.0%
'g5g5x'	0.0%	0.1%
'g5g6g6'	0.0%	0.3%
'g5g6n3'	0.0%	0.1%
'g5g6n4'	0.0%	0.1%
'g5g6x'	0.0%	0.6%
'g5n34'	0.0%	0.1%
'g5n3n3'	0.0%	0.0%
'g5n3n4'	0.0%	0.0%
'g5n3x'	0.0%	0.1%
'g5n4n4'	0.0%	0.0%
'g5n4x'	0.0%	0.1%
'g5xx'	0.0%	1.0%
'g6g6g6'	0.2%	0.2%
'g6g6n3'	0.1%	0.1%
'g6g6n4'	0.1%	0.1%
'g6g6x'	0.2%	0.5%
'g6n34'	0.2%	0.3%
'g6n3n3'	0.0%	0.0%
'g6n3n4'	0.0%	0.0%
'g6n3x'	0.0%	0.2%
'g6n4n4'	0.0%	0.0%
'g6n4x'	0.0%	0.2%
'g6xx'	0.2%	1.2%
'n234'	0.4%	1.4%
'n345'	0.4%	1.4%
'n34n4'	0.0%	0.0%
'n34x'	0.0%	0.3%
'n3n34'	0.0%	0.0%
'n3n3n3'	0.0%	0.0%
'n3n3n4'	0.0%	0.0%
'n3n3x'	0.0%	0.0%
'n3n4n4'	0.0%	0.0%
'n3n4x'	0.0%	0.0%
'n3xx'	0.0%	0.3%
'n4n4n4'	0.0%	0.0%
'n4n4x'	0.0%	0.0%
'n4xx'	0.0%	0.3%
'xxx'	0.4%	3.5%

'g2g6n3'	0.0%	0.0%
'g2g6n4'	0.0%	0.0%
'g2g6x'	0.2%	0.3%
'g2n34'	0.1%	0.1%
'g2n3n3'	0.0%	0.0%
'g2n3n4'	0.0%	0.0%
'g2n3x'	0.0%	0.1%
'g2n4n4'	0.0%	0.0%
'g2n4x'	0.0%	0.1%
'g2xx'	0.2%	0.7%
'g3g3g3'	0.0%	0.0%
'g3g3g5'	0.0%	0.0%
'g3g3g6'	0.0%	0.0%
'g3g3n3'	0.0%	0.0%
'g3g3n4'	0.0%	0.0%
'g3g3x'	0.0%	0.0%
'g3g56'	0.4%	0.0%
'g3g5g5'	0.0%	0.0%
'g3g5g6'	0.1%	0.0%
'g3g5n3'	0.0%	0.0%
'g3g5n4'	0.0%	0.0%
'g3g5x'	0.1%	0.0%
'g3g6g6'	0.1%	0.0%
'g3g6n3'	0.0%	0.0%
'g3g6n4'	0.0%	0.0%
'g3g6x'	0.2%	0.0%
'g3n34'	0.0%	0.0%
'g3n3n3'	0.0%	0.0%
'g3n3n4'	0.0%	0.0%
'g3n3x'	0.0%	0.0%
'g3n4n4'	0.0%	0.0%
'g3n4x'	0.0%	0.0%
'g3xx'	0.4%	0.0%
'g456'	2.0%	-6.0%
'g56g6'	0.2%	0.3%
'g56n3'	0.0%	0.0%
'g56n4'	0.0%	0.0%
'g56x'	0.3%	0.5%
'g5g56'	0.2%	0.6%
'g5g5g5'	0.0%	0.0%
'g5g5g6'	0.0%	0.1%
'g5g5n3'	0.0%	0.0%
'g5g5n4'	0.0%	0.0%
'g5g5x'	0.0%	0.1%
'g5g6g6'	0.1%	0.1%
'g5g6n3'	0.0%	0.1%
'g5g6n4'	0.0%	0.1%
'g5g6x'	0.2%	0.3%
'g5n34'	0.0%	0.1%
'g5n3n3'	0.0%	0.0%
'g5n3n4'	0.0%	0.0%
'g5n3x'	0.0%	0.1%
'g5n4n4'	0.0%	0.0%
'g5n4x'	0.0%	0.1%
'g5xx'	0.3%	0.8%
'g6g6g6'	0.0%	-0.1%
'g6g6n3'	0.0%	0.0%
'g6g6n4'	0.0%	0.0%
'g6g6x'	0.1%	-0.1%
'g6n34'	0.1%	0.0%
'g6n3n3'	0.0%	0.0%
'g6n3n4'	0.0%	0.0%
'g6n3x'	0.0%	0.0%
'g6n4n4'	0.0%	0.0%
'g6n4x'	0.0%	0.0%
'g6xx'	0.2%	-0.1%
'n234'	0.2%	0.5%
'n345'	0.2%	0.5%
'n34n4'	0.0%	0.0%
'n34x'	0.1%	0.1%
'n3n34'	0.0%	0.0%
'n3n3n3'	0.0%	0.0%
'n3n3n4'	0.0%	0.0%
'n3n3x'	0.0%	0.0%
'n3n4n4'	0.0%	0.0%
'n3n4x'	0.0%	0.0%
'n3xx'	0.1%	0.0%
'n4n4n4'	0.0%	0.0%
'n4n4x'	0.0%	0.0%
'n4xx'	0.1%	0.0%
'xxx'	0.6%	1.0%
