# Supplementary figures and images for: Rational design of 13C-labeling experiments for metabolic flux analysis in mammalian cells
Source: BMC Syst Biol. 2012 May 16;6:43. doi: 10.1186/1752-0509-6-43 (PMC3490712; doi:10.1186/1752-0509-6-43)

# A

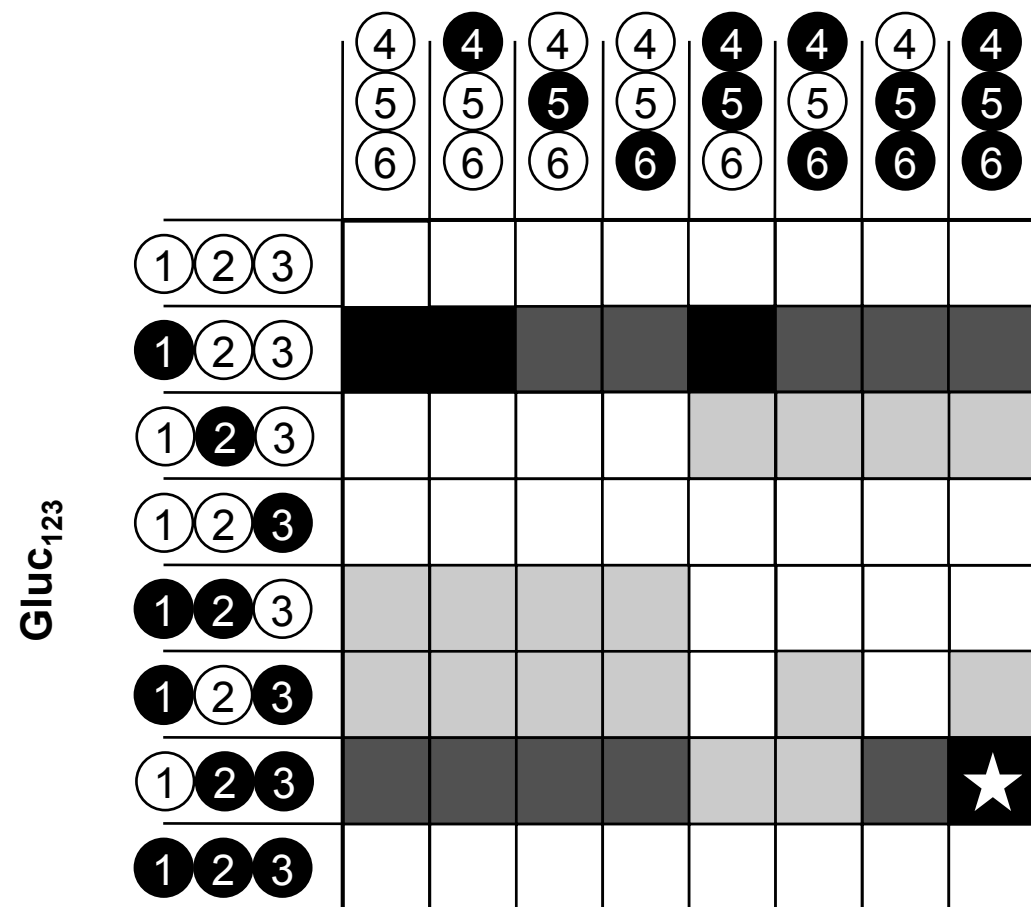

|                                                                                     |            |
|-------------------------------------------------------------------------------------|------------|
| 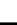 | 0 – 10%    |
| 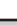 | 10 – 12.5% |
| 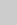 | 12.5 – 15% |
| 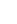 | 15% +      |
| 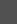 | Optimal    |

# B

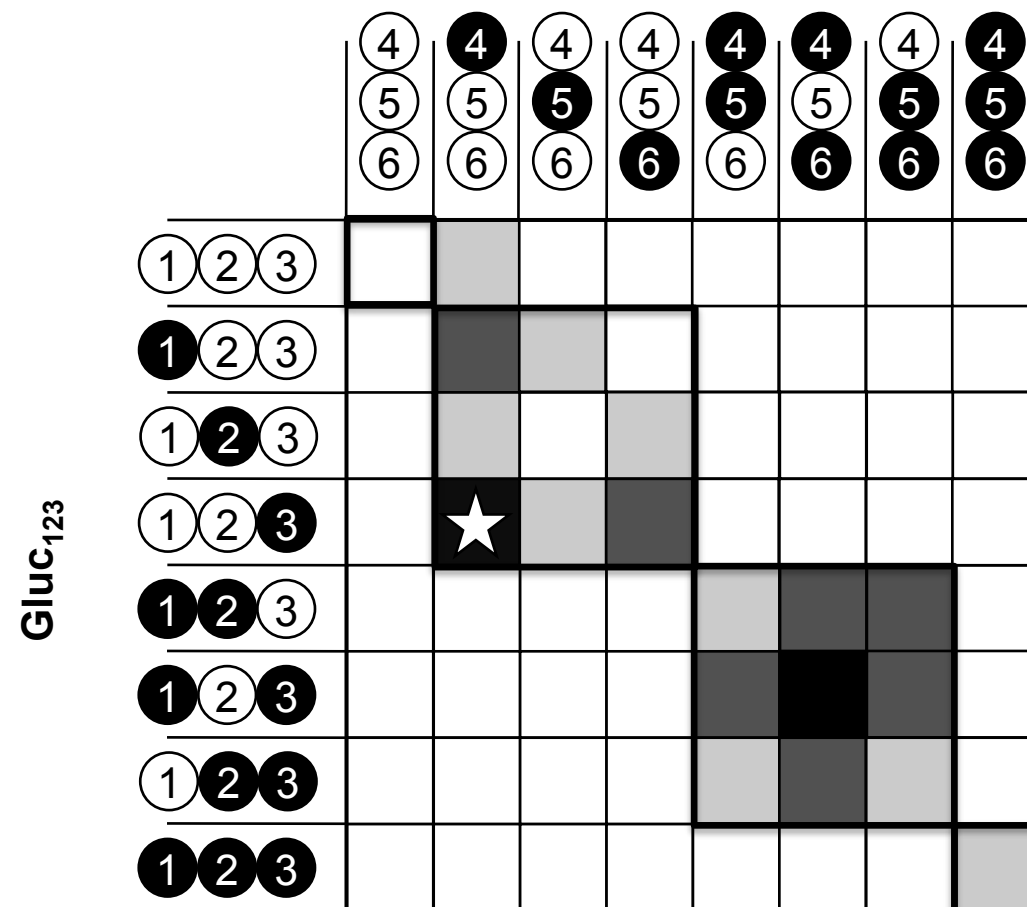

|                                                                                     |         |
|-------------------------------------------------------------------------------------|---------|
| 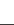 | 0 – 6%  |
| 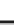 | 6 – 8%  |
| 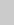 | 8 – 10% |
| 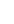 | 10% +   |
| 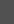 | Optimal |

Supplement: Additional file 3 — – Sensitivities of tracers to the oxPPP and PC fluxes. Title: Sensitivities of tracers to the oxPPP and PC fluxes. Description: Maximum sensitivity for glucose tracers with respect to (A) oxPPP flux and (B) PC flux. [file 1752-0509-6-43-S3.pdf]

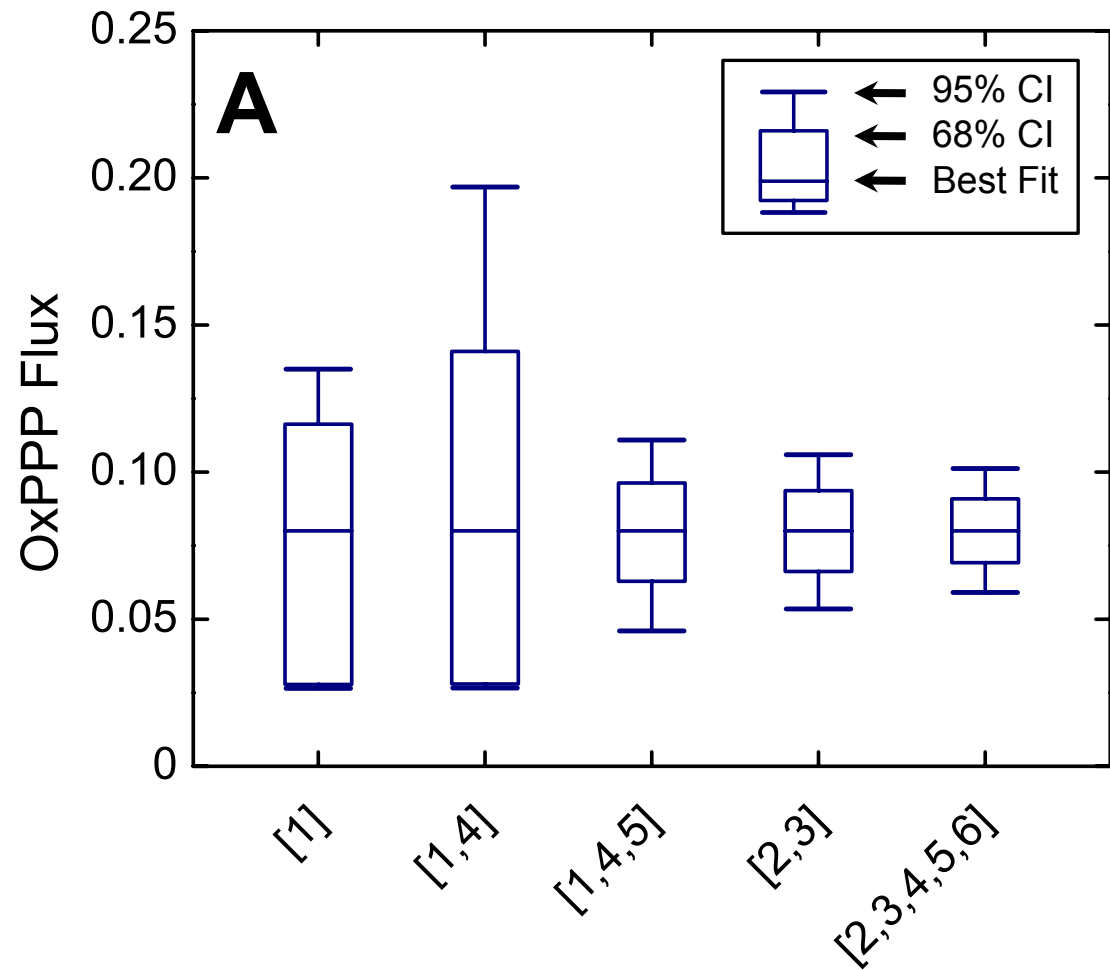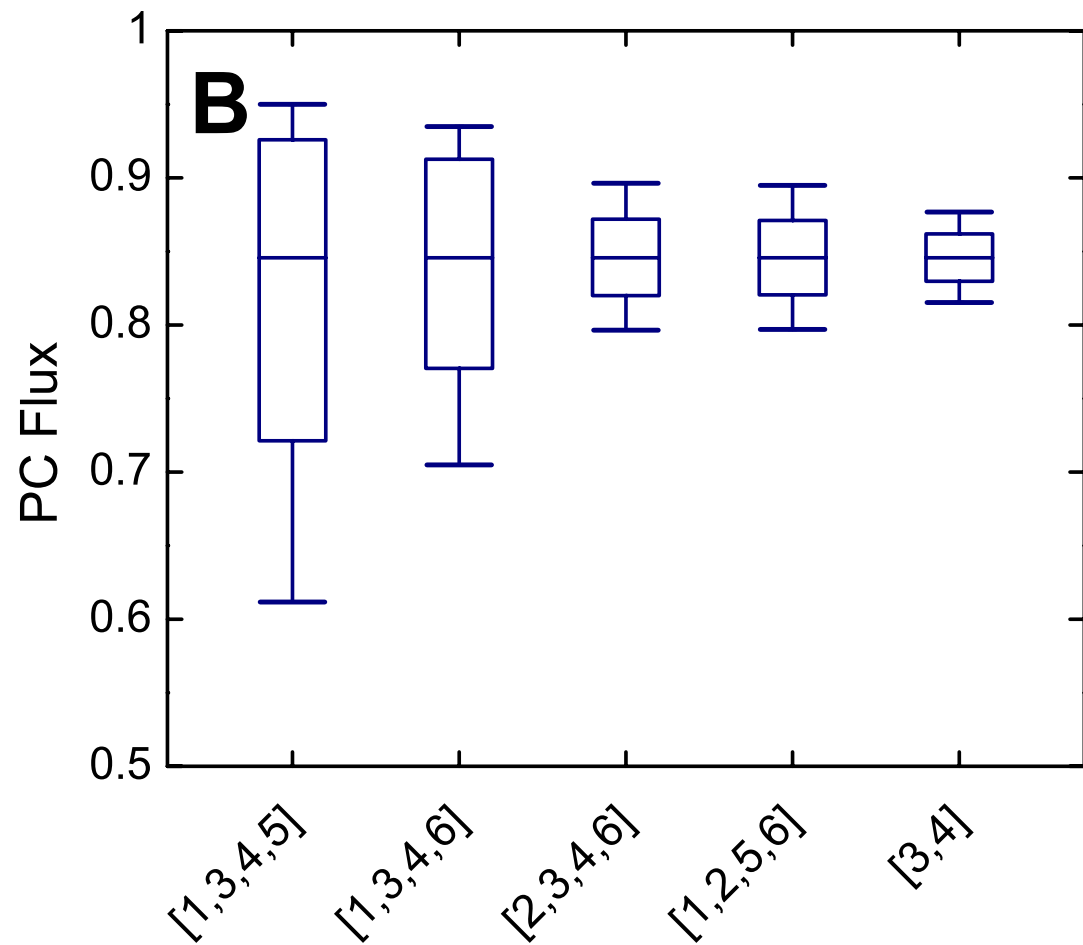

Supplement: Additional file 4 — – Confidence intervals of oxPPP and PC fluxes for various glucose tracers. Title: Confidence intervals of oxPPP and PC fluxes for various glucose tracers. Description: Representative confidence intervals for glucose tracers for (A) oxPPP flux and (B) PC flux. [file 1752-0509-6-43-S4.pdf]

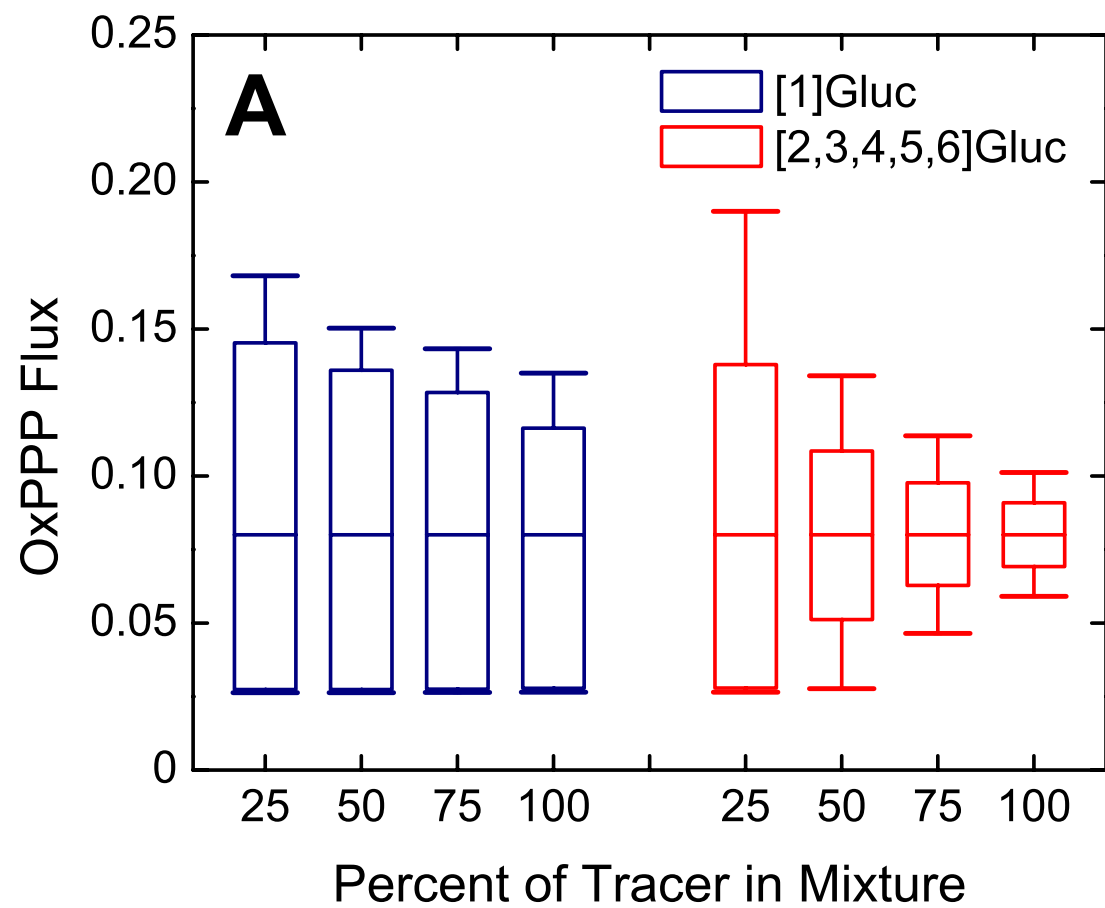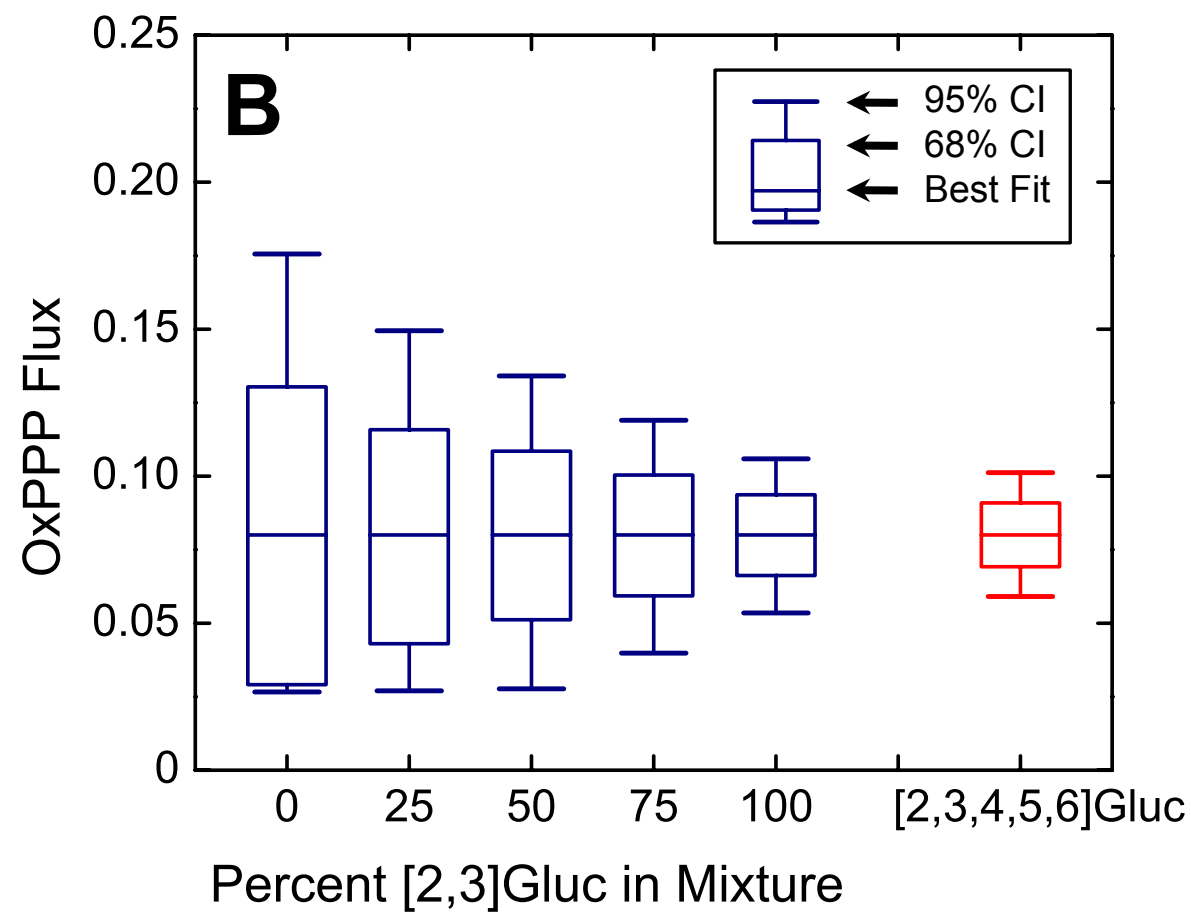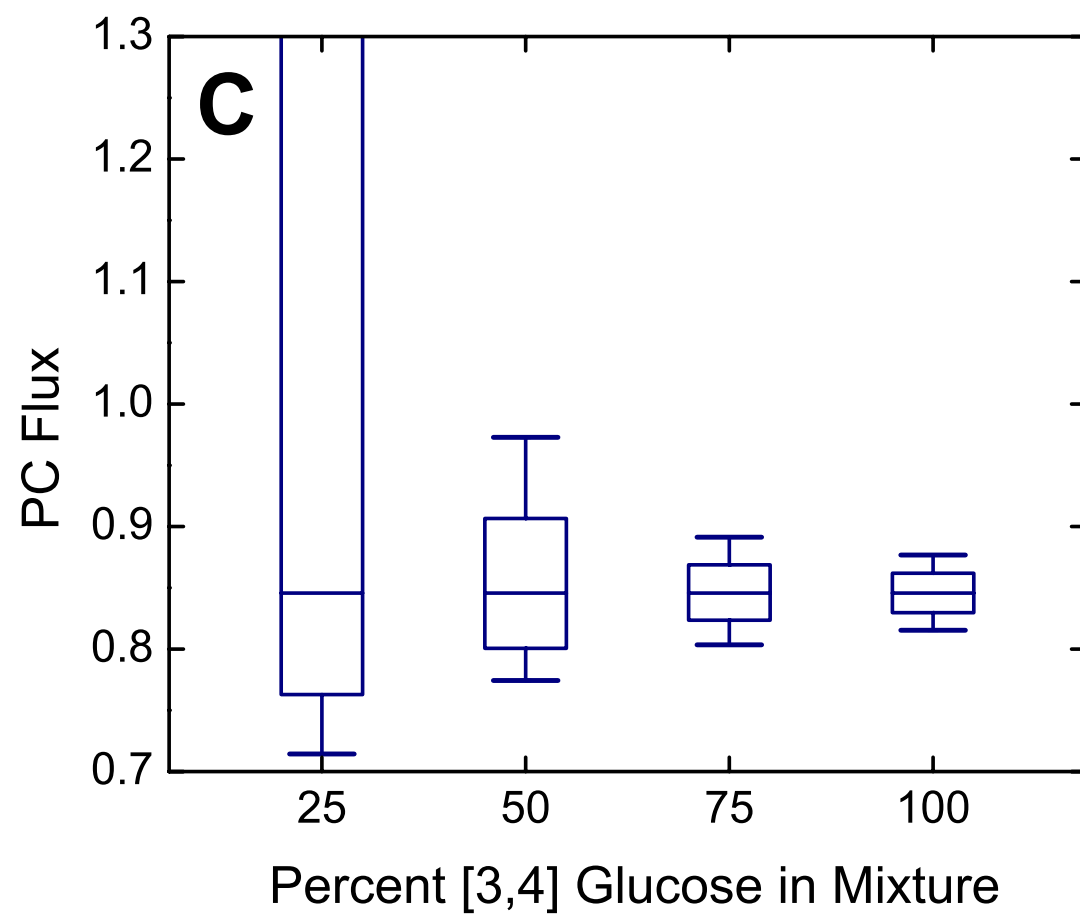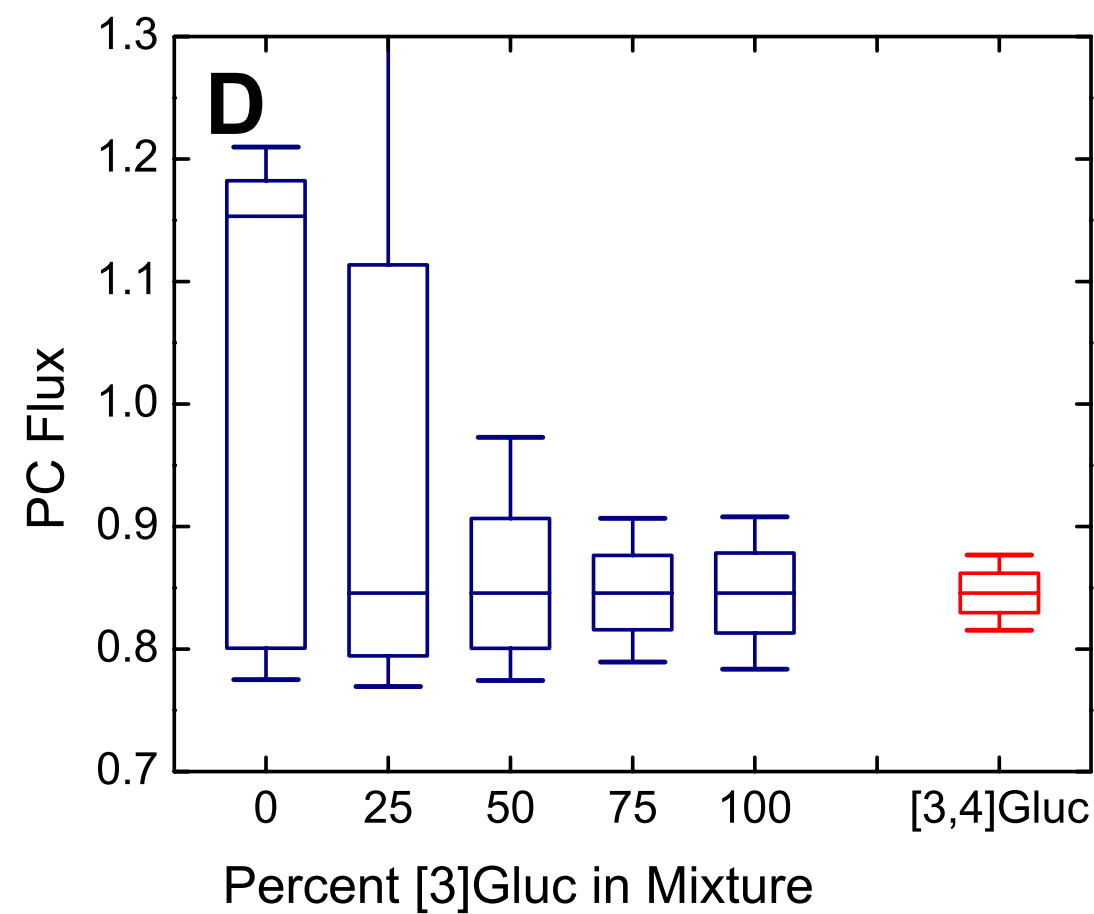

Supplement: Additional file 5 — – Effect of mixtures on oxPPP and PC confidence intervals. Title: Effect of mixtures on oxPPP and PC confidence intervals. Description: Mixture effects of unlabeled glucose tracer for optimal oxPPP and PC tracers (A & C). Comparison of commercially available tracers mixtures versus the custom synthesized counterparts for oxPPP and PC fluxes (B & D). [file 1752-0509-6-43-S5.pdf]

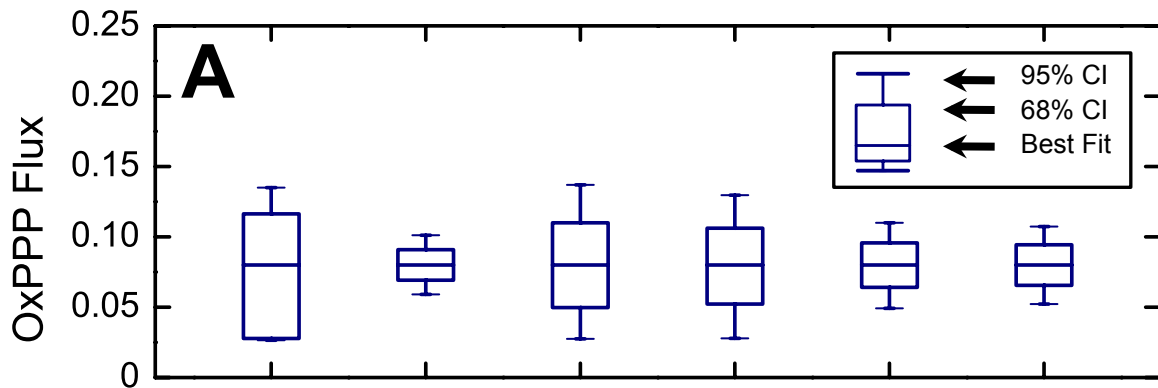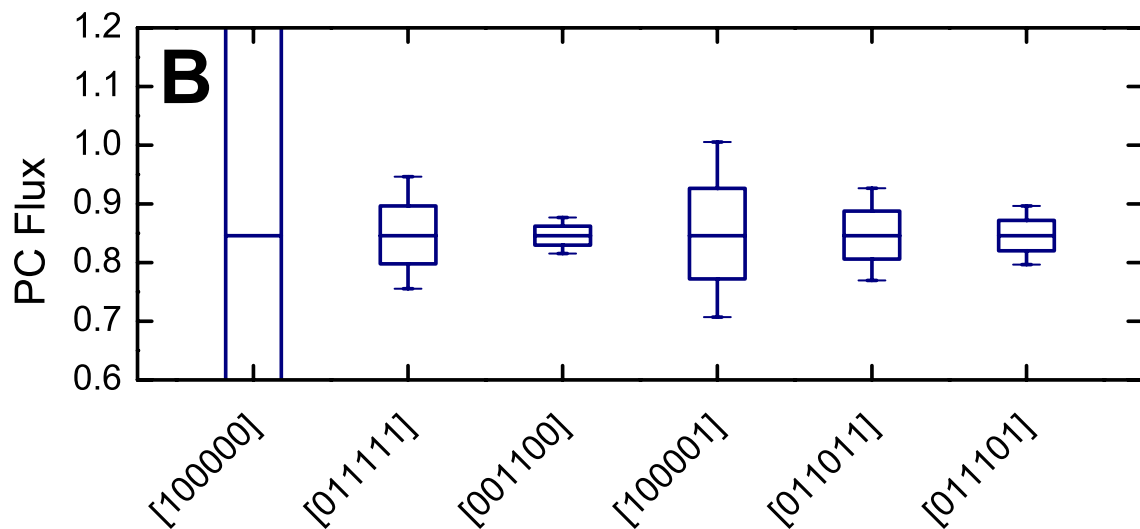

Supplement: Additional file 6 — – Resolution of oxPPP and PC fluxes with a single tracer. Title: Resolution of oxPPP and PC fluxes with a single tracer. Description: Comparison of confidence intervals for (A) oxPPP flux and (B) PC flux for optimal tracers ([1-13C]glucose, [2,3,4,5,6-13C]glucose, and [3,4-13C]glucose), and others. [file 1752-0509-6-43-S6.pdf]

[1] Glucose

[2,3,4,5,6] Glucose

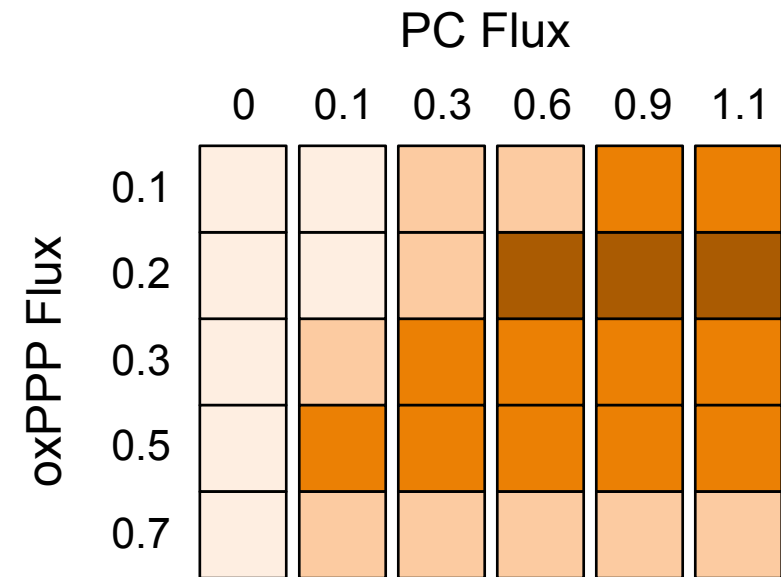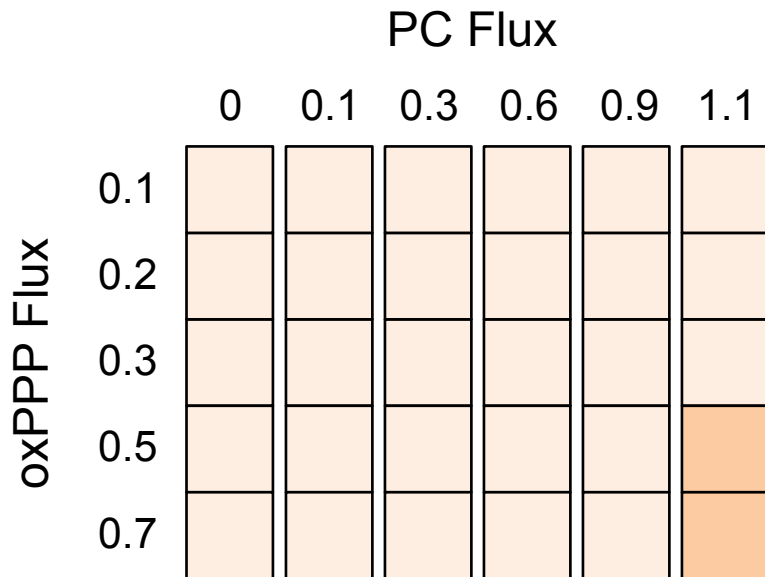

**Range of  
Conf. Interval**

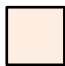

0 – 0.05

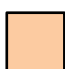

0.05 – 0.10

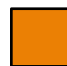

0.10 – 0.15

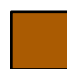

0.15 – 0.20

Supplement: Additional file 7 — – Comparison of [1-13C]glucose and [2,3,4,5,6-13C]glucose for oxPPP resolution. Title: Comparison of [1-13C]glucose and [2,3,4,5,6-13C]glucose for oxPPP resolution. Description: Range of confidence intervals for [1-13C]glucose and [2,3,4,5,6-13C]glucose over various combinations of oxPPP and PC flux values. [file 1752-0509-6-43-S7.pdf]

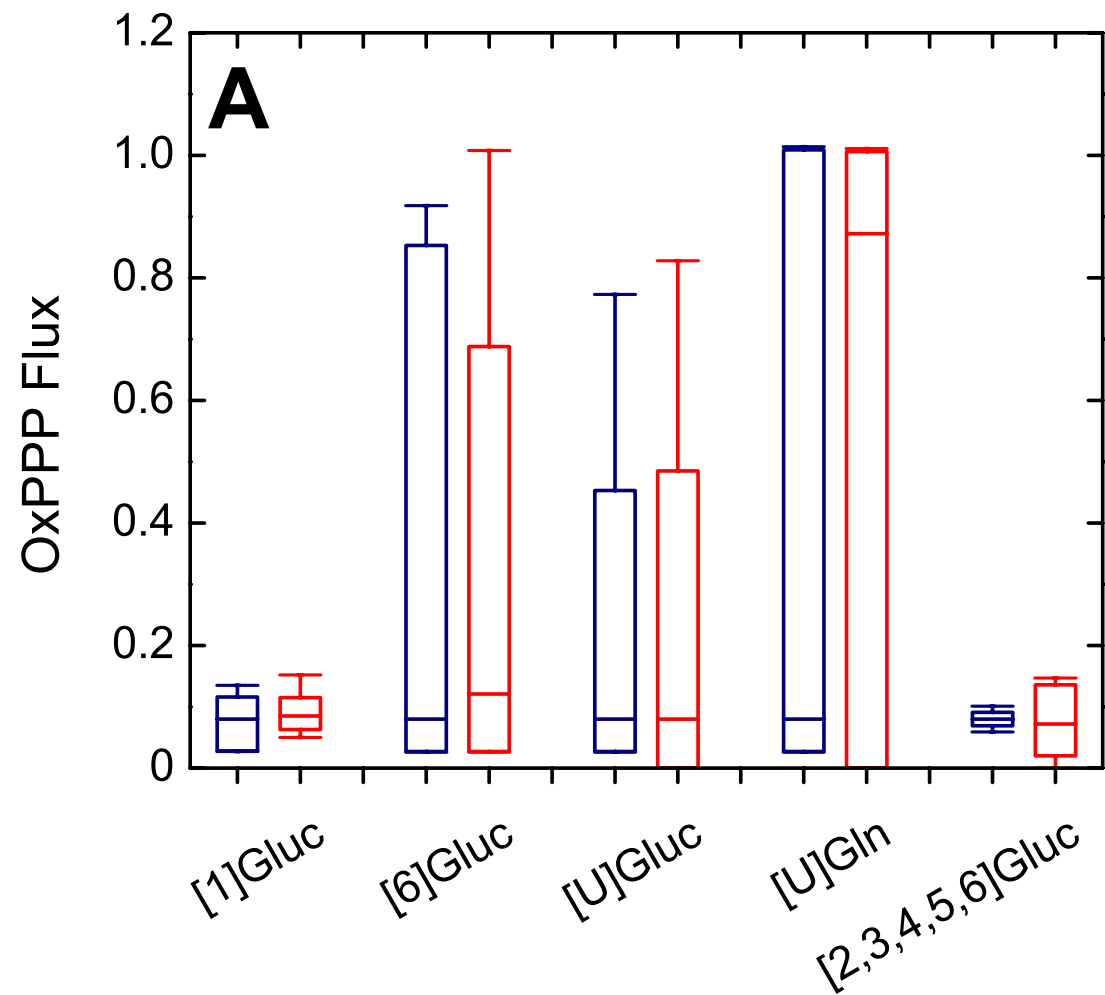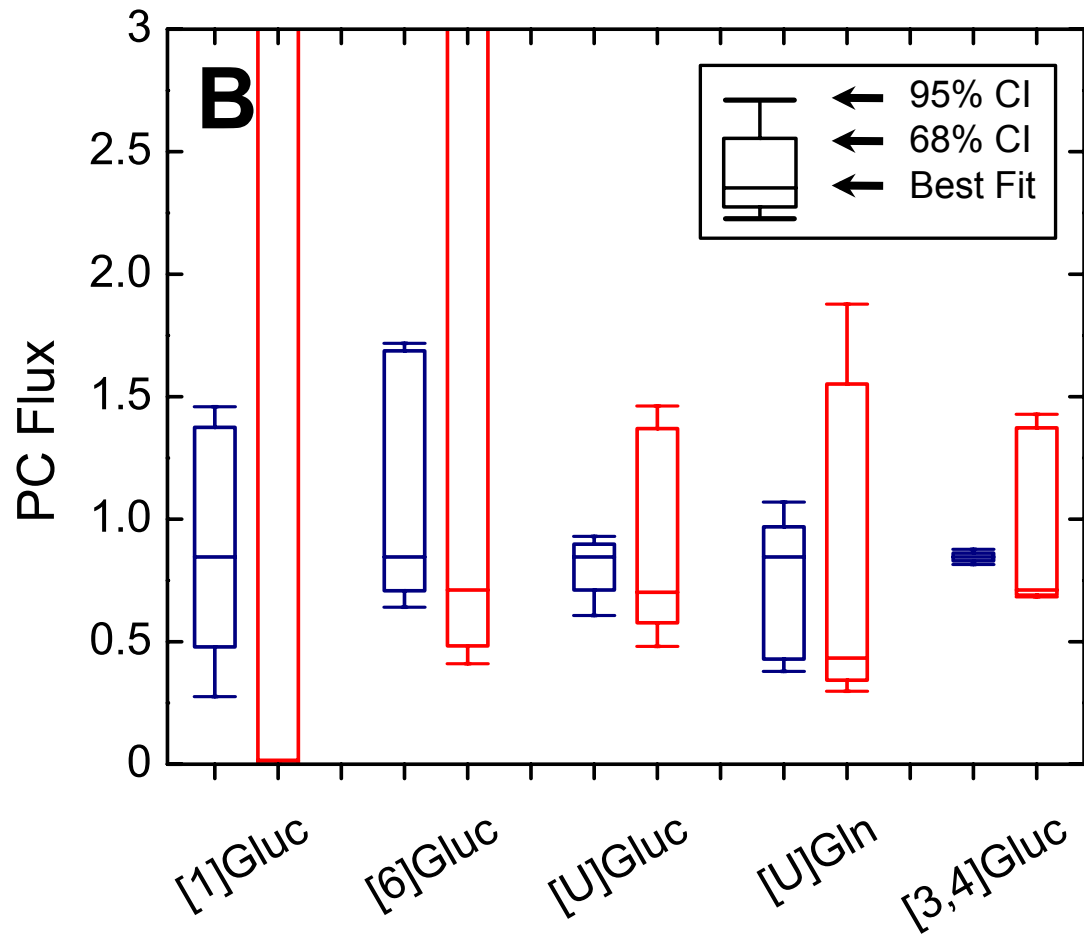

Supplement: Additional file 8 — – Comparison of reaction reversibilities on confidence intervals for oxPPP and PC fluxes. Title: Comparison of reaction reversibilities on confidence intervals for oxPPP and PC fluxes. Description: Simulation of (A) oxPPP and (B) PC confidence intervals for network model without reversible fluxes (blue bars) and with reversible reactions included (red bars). For simulations including reversible reactions, the assumed exchange fluxes and normalized flux values were: transketolase (0.08), transaldolase (0.08), and malate dehydrogenase (1.1). [file 1752-0509-6-43-S8.pdf]
